# Supplementary material for: Next generation AAV-F capsid gene therapy rescues disease pathology in a model of pyruvate dehydrogenase complex deficiency
Source: Mol Ther Adv. 2026 Jun 15;34(3):201781. doi: 10.1016/j.omta.2026.201781 (PMC13355638; doi:10.1016/j.omta.2026.201781)
Supplement: Document S1. Figures S1–S7, Table S1, and supplemental methods [file mmc1.pdf]

OMTA, Volume *34*

Supplemental information

Next generation AAV-F capsid gene therapy rescues disease pathology in a model of pyruvate dehydrogenase complex deficiency

Anna Keegan, Özge Çetin, Ellie M. Chilcott, Juan Antinao Diaz, Simon Eaton, Simon N. Waddington, John R. Counsell, Shamima Rahman, and Rajvinder Karda

Figure S1

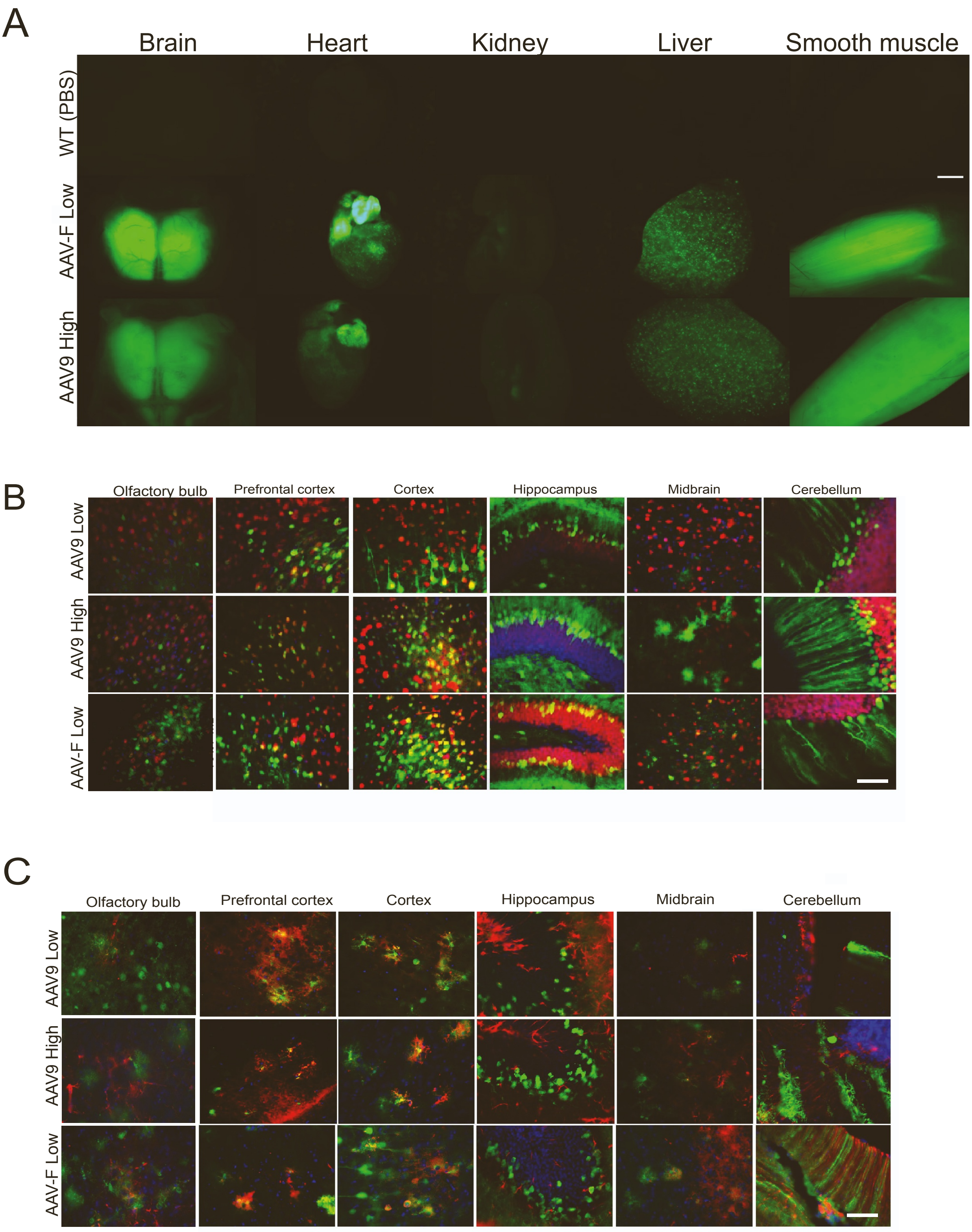

Figure S1. GFP Biodistribution of AAV9 and AAV-F. (A) Representative images of *ex vivo* GFP after neonatal ICV injections of AAV-F and AAV9. *Ex-vivo* GFP analysis at 100 days post injection. Scale bars shown in each image (2mm). (B) Transduction of neurons following the administration of AAV9 low dose and high dose and AAV-F low dose. Co-immunofluorescence with neuronal marker NeuN (red), GFP (green) and nuclear stain, DAPI (blue). Representative images of olfactory bulb, prefrontal cortex, cortex (reused from Figure 1D), hippocampus, midbrain and cerebellum. Images were taken at 40X magnification. Scale bar = 100µm. (C) Transduction of astrocytes following the administration of AAV9 low dose and high dose and AAV-F low dose. Co-immunofluorescence with astrocyte marker GFAP (red), GFP (green) and nuclear stain, DAPI (blue). Representative images of olfactory bulb, prefrontal cortex, cortex (reused from Figure 1F), hippocampus, midbrain and cerebellum. Images were taken at 40X magnification. Scale bar = 100µm.

Figure S2

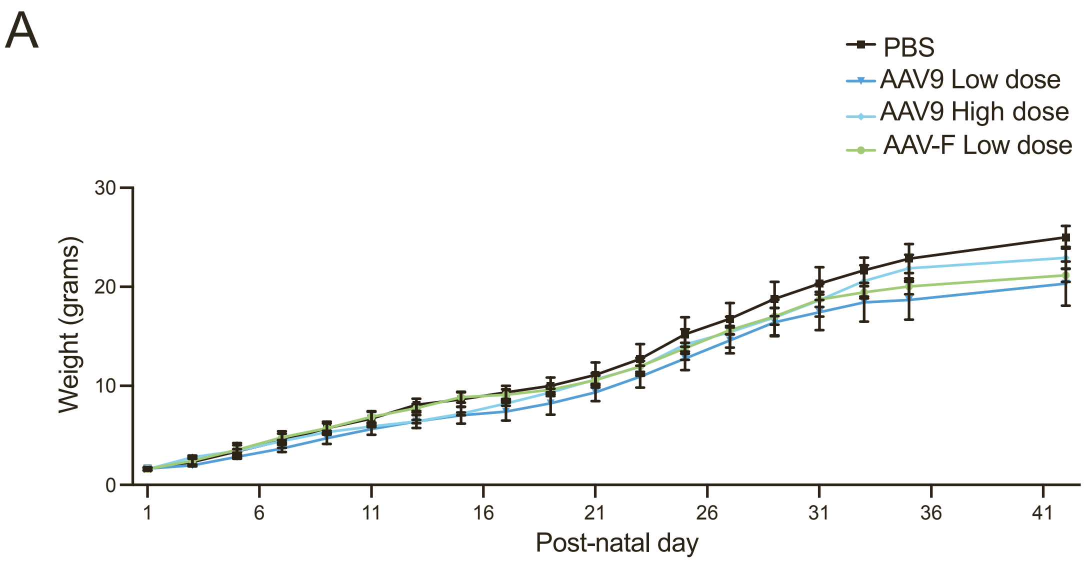

Figure S2. WT toxicology study of therapeutic vector following ICV delivery of AAV9 and AAV-F.
(A) Weights of WT treated mice shown as mean ± SEM. One-way ANOVA multiple comparison.

Figure S3

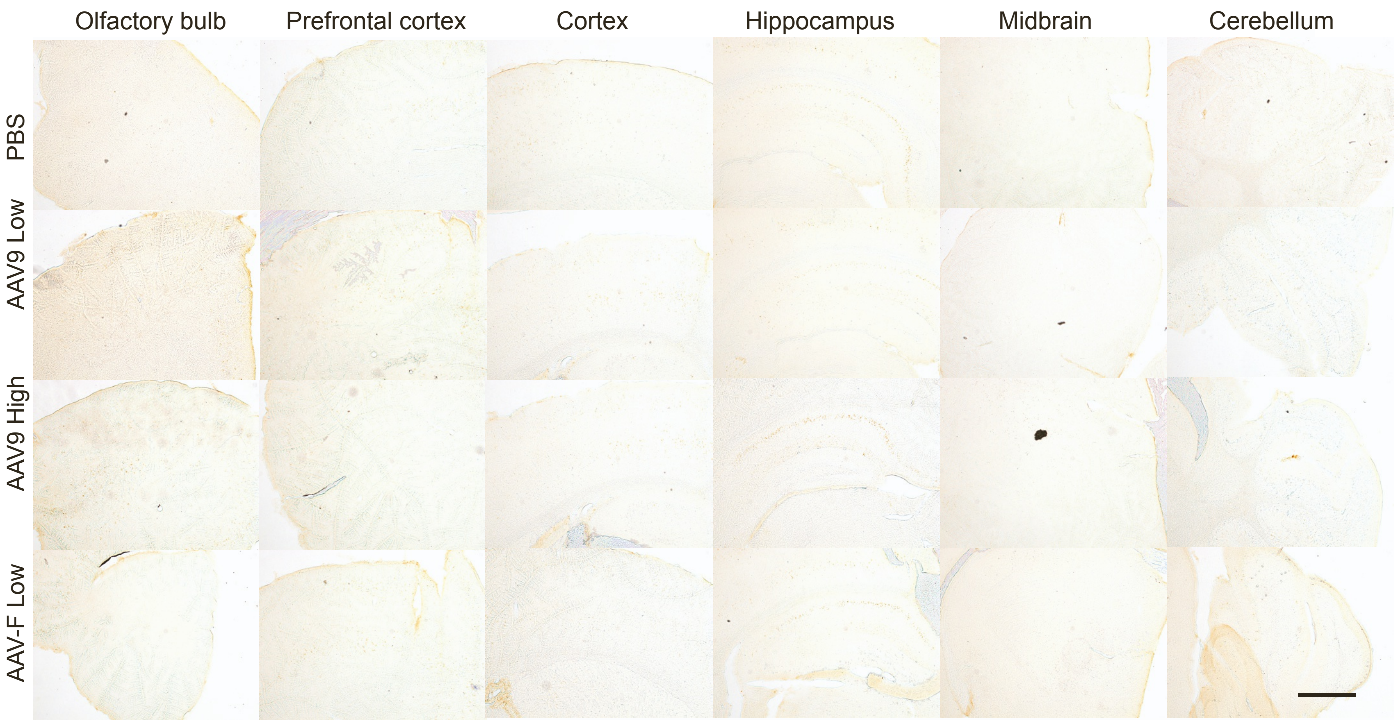

Figure S3. CD68 immunohistochemical assessment of treated WT brain samples. Representative images of CD68 expression in WT mice treated with PBS, AAV9 low dose, AAV9 high dose and AAV-F low dose for prefrontal cortex, cortex, hippocampus, midbrain and cerebellum. Images were taken at 40X magnification. Scale bar = 100µm.

Figure S4

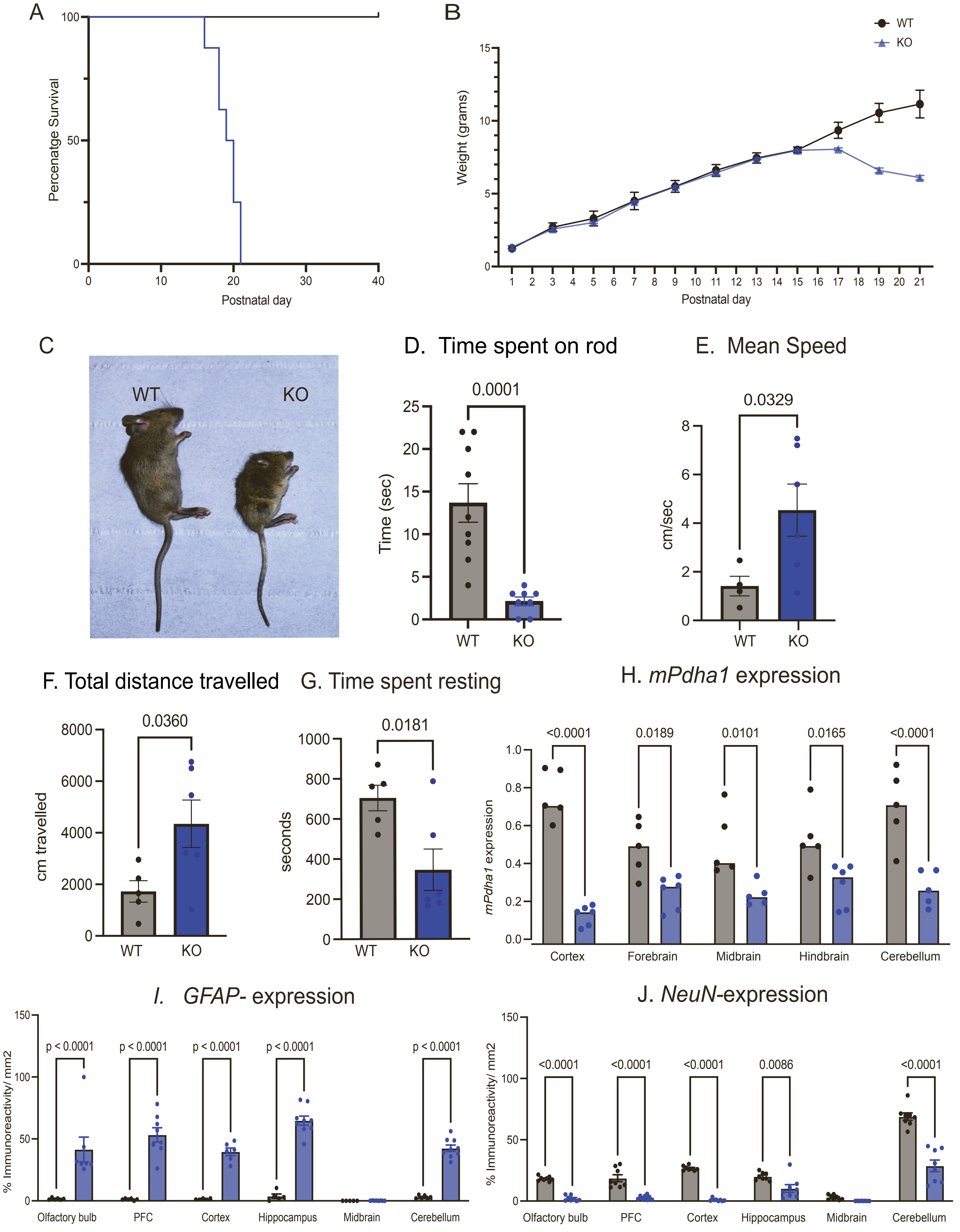
A
100
50
0
Percenatge Survival
0
10
20
30
40
Postnatal day
B
WT
KO
15
10
5
0
Weight (grams)
1
2
3
4
5
6
7
8
9
10
11
12
13
14
15
16
17
18
19
20
21
Postnatal day
C
WT
KO
D. Time spent on rod
0.0001
25
20
15
10
5
0
Time (sec)
WT
KO
E. Mean Speed
0.0329
8
6
4
2
0
cm/sec
WT
KO
F. Total distance travelled
0.0360
8000
6000
4000
2000
0
cm travelled
WT
KO
G. Time spent resting
0.0181
1000
800
600
400
200
0
seconds
WT
KO
H. *mPdha1* expression
<0.0001
0.0189
0.0101
0.0165
<0.0001
1.0
0.8
0.6
0.4
0.2
0.0
mPdha1 expression
Cortex
Forebrain
Midbrain
Hindbrain
Cerebellum
I. *GFAP*- expression
150
100
50
0
% Immunoreactivity/ mm2
p < 0.0001
p < 0.0001
p < 0.0001
p < 0.0001
p < 0.0001
Olfactory bulb
PFC
Cortex
Hippocampus
Midbrain
Cerebellum
J. *NeuN*-expression
150
100
50
0
% Immunoreactivity/ mm2
<0.0001
<0.0001
<0.0001
0.0086
<0.0001
Olfactory bulb
PFC
Cortex
Hippocampus
Midbrain
Cerebellum

Figure S4

Figure S4. Characterisation of untreated PDHc deficient mice. (A) Kaplan-Meier survival curve of KO mice compared to healthy controls (n=10). (B) Weights of KO mice compared to healthy controls. (C) Image of PDHc deficient mouse (right) alongside WT litter mate (left). (D) Rotarod assessment plotted as time (seconds) spent on the accelerating rod. *Pdha1* KO mice had a significantly lower latency to fall compared to healthy age matched littermates at postnatal 16. (E) Open field test at postnatal 16 showing mean speed over 15 minutes. Welch's t-test ± SEM. (n=10). (F) Open field test showing total distance travelled in 15 minutes. Welch's t-test ± SEM. (G) Open field test quantifying the resting time in 15 minutes. Welch's t-test ± SEM. (H) Fold change of *Pdha1* gene expression to *Gapdh* in *Pdha1* KO mice compared to WT littermates at time of collection (P18-21). (I) GFAP expression in *Pdha1* KO brain compared to WT littermates showing an upregulation of astrocytes throughout the brain. (J) NeuN expression in *Pdha1* KO brain compared to WT littermates showing extensive neuronal loss.

Figure S5

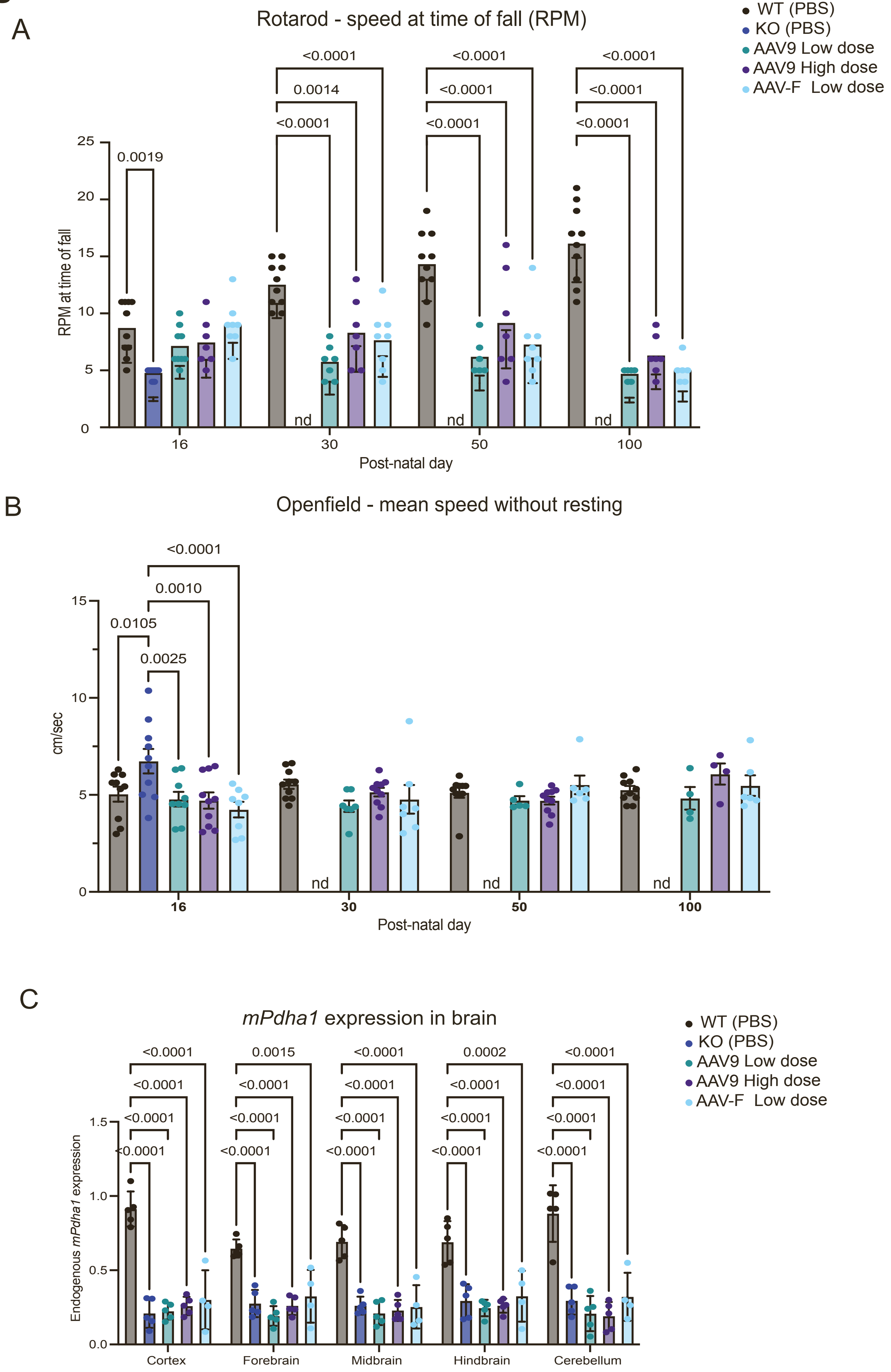
A
Rotarod - speed at time of fall (RPM)
RPM at time of fall
Post-natal day
16
30
50
100
0.0019
<0.0001
0.0014
nd
WT (PBS)
KO (PBS)
AAV9 Low dose
AAV9 High dose
AAV-F Low dose
B
Openfield - mean speed without resting
cm/sec
0.0105
0.0010
0.0025
C
mPdha1 expression in brain
Endogenous mPdha1 expression
Cortex
Forebrain
Midbrain
Hindbrain
Cerebellum
0.0002
0.0015

Figure S5

D

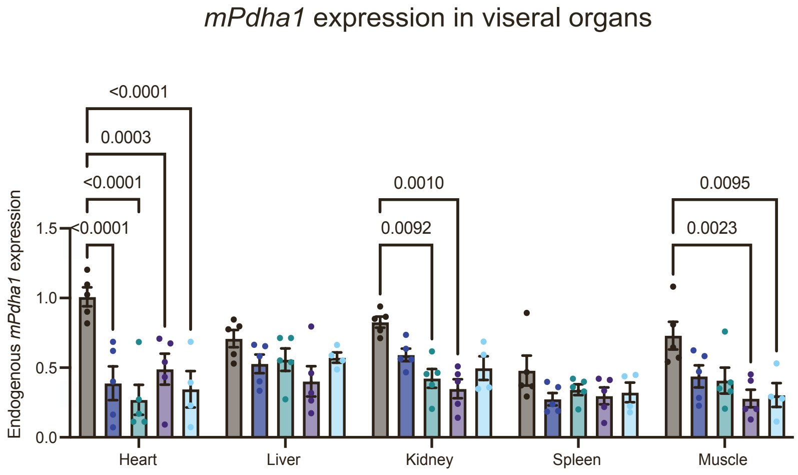

Figure S5. Further assessment of PBS-treated WT controls, and PBS, AAV9 low dose and high dose and AAV-F low dose treated KO's. (A) Behavioural rotarod assessment, showing speed on the time of fall. One-way ANOVA, Tukey's multiple comparison ± SEM. Not done (nd). (B) Open field test showing mean speed without resting in 15 minutes. One-way ANOVA, Tukey's multiple comparison ± SEM. Not done (nd). (C) Endogenous *Pdha1* expression in discrete regions of the brains of the treated KO mice. One-way ANOVA, Tukey's multiple comparison ± SEM. (D) Endogenous *Pdha1* expression in visceral organs of the treated KO mice. One-way ANOVA, Tukey's multiple comparison ± SEM.

Figure S6

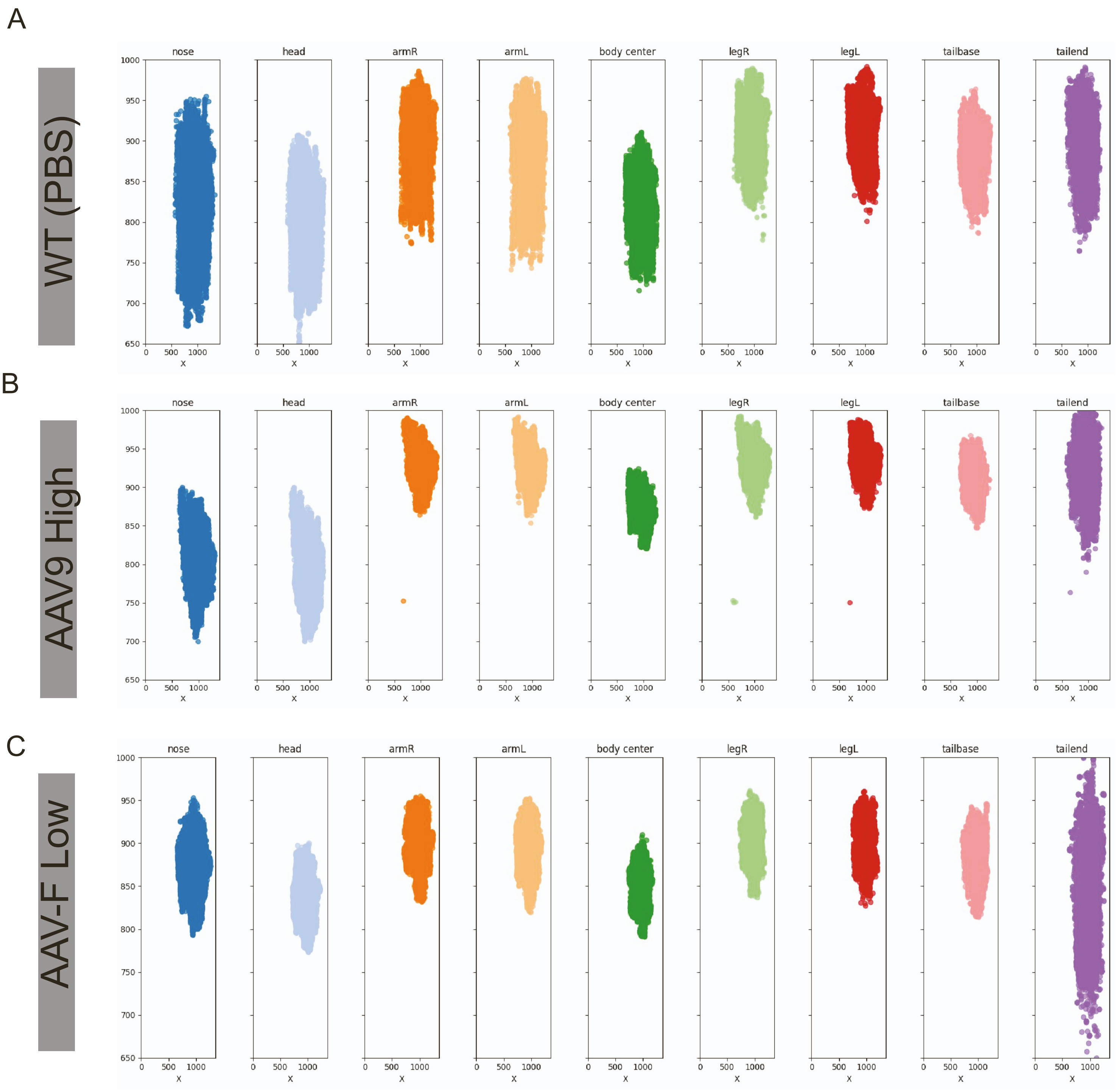

Figure S6. Individual body part assessment using DLC. (A) This plot visualises the movement path of a specific body part across time. Each point represents the X–Y position in the video frame, allowing quantification of locomotor patterns and spatial exploration. Targeted body part separated trajectory plots representing the PBS-treated control group. In all graphs, each body part is consistently color-coded as follows: nose (blue), head (light blue), right arm (dark orange), left arm (light orange), body centre (dark green), right leg (light green), left leg (red), tail base (pink), and tail end (purple). (B) Targeted body part separated trajectory plots representing AAV9 high dose treated KO mice show a change in individual body part movements compared to control mice. (C) Targeted body part separated trajectory plots representing AAV-F low dose treated KO mice show a change in individual body part movements compared to control mice.

Figure S7

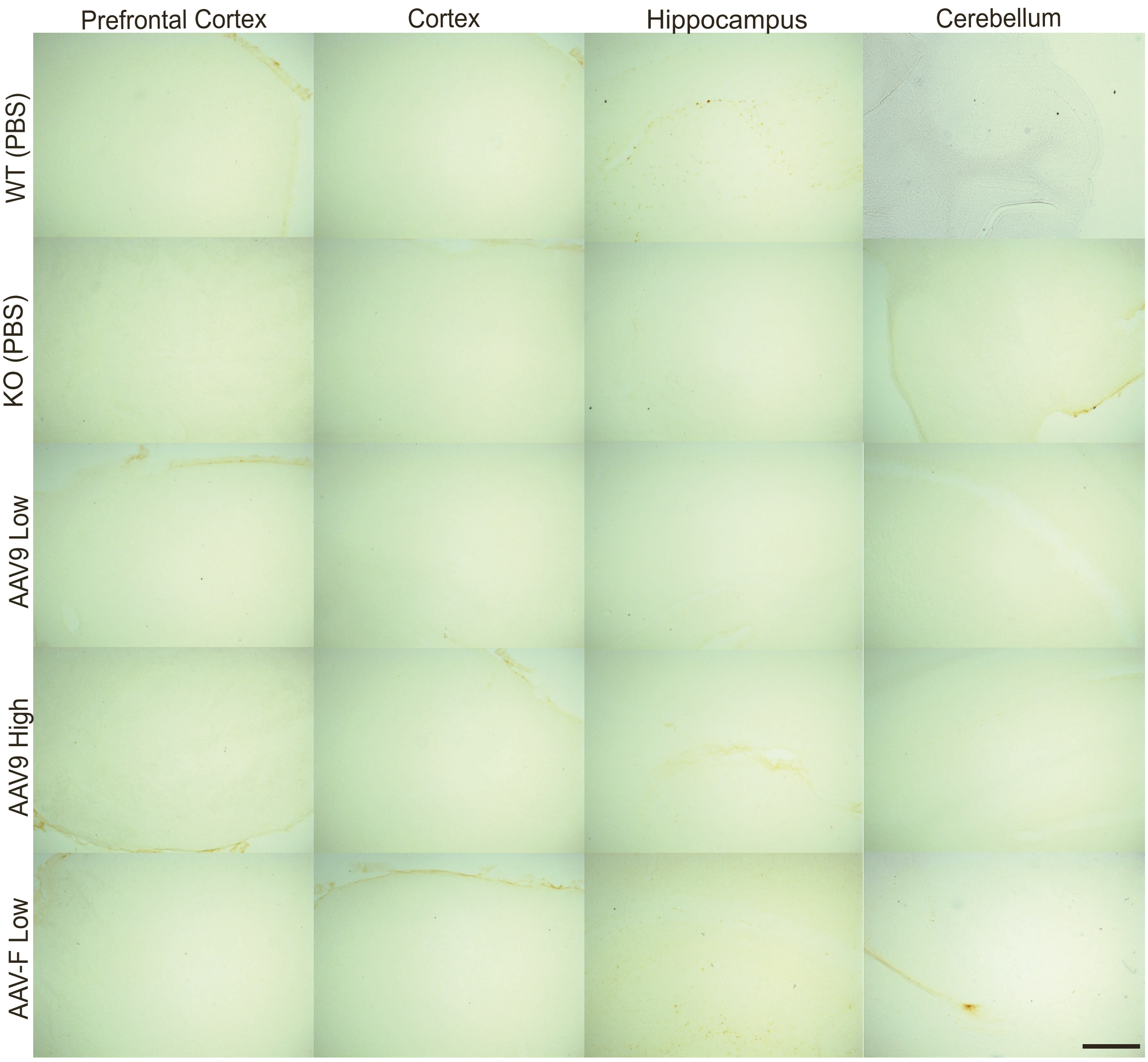

Figure S7. CD68 immunohistochemical assessment of treated brain samples. Representative images of CD68 expression in KO-treated mice for prefrontal cortex, cortex, hippocampus, and cerebellum. Images were taken at 40X magnification. Scale bar = 100μm.

Supplemental Methods

DLC Code

```
position_changes = {}
for group, dfs in group_data.items():
position_changes[group] = {}
for part in body_parts:
change_list = []
for df in dfs:
# Check if both x and y columns for the body part exist
if (part, 'x') in df.columns and (part, 'y') in df.columns:
x_series = df[(part, 'x')]
y_series = df[(part, 'y')]
# Keep only valid (non-zero) x and y values
valid_mask = (x_series != 0) & (y_series != 0)
x_valid = x_series[valid_mask]
y_valid = y_series[valid_mask]
# Need at least 2 valid points to compute displacement
if len(x_valid) >= 2 and len(y_valid) >= 2:
dx = np.diff(x_valid)
dy = np.diff(y_valid)
step_distances = np.sqrt(dx**2 + dy**2)
total_movement = np.sum(step_distances)
change_list.append(total_movement)
else:
print(f"Not enough valid data for {part} in a file from group {group}.")
else:
print(f"Missing columns {(part, 'x')} or {(part, 'y')} in a file from group {group}.")
if change_list:
position_changes[group][part] = change_list
```

Supplemental Tables

Table S1. Table of primers, probes and sequences used in this study.

**Primer Target**		**Sequence 5'-3'**
mPdha1 Genotyping	For	CGT CTG TTGAGA GAG CAG CA
mPdha1 Genotyping	Rev	CGC ACA AGATAT CCA TTC CA
hGFAP-Cre Genotyping	For	ACT CCT TCATAA AGC CCT
hGFAP-Cre Genotyping	Rev	ATC ACT CGT TGC ATC GAC CG
hPDHA1	For	GGAGGATGGGCTCAAATACTAC
hPDHA1	Rev	GACCATCACACAAGTGACAGA
hPDHA1	Probe	ACGCCGAATGGAGTTGAAAGCAGA
mPdha1	For	ATTCCTGGACTCAGGGTAGAT
mPdha1	Rev	CCCTTACCAGACCTGCAATAG
mPdha1	Probe	TATCTTGTGCGTCCGAGAGGCAAC
mGapdh	For	ACGGCAAATTCAACGGCAC
mGapdh	Rev	TAGTGGGGTCTCGCTCCTGG
mGapdh	Probe	TTGTCATCAACGGGAAGCCCATCA

Supplemental Videos

Video S1. Videos of treated KO *Pdha1* mice labeled with body parts: Control mouse.

Video S2. Videos of treated KO Pdha1 mice labeled with body parts: AAV9 high dose treated mouse.

Video S3. Videos of treated KO Pdha1 mice labeled with body parts: AAV-F low dose treated mouse.
