# Supplementary material for: Next generation AAV-F capsid gene therapy rescues disease pathology in a model of pyruvate dehydrogenase complex deficiency
Source: Mol Ther Adv. 2026 Jun 15;34(3):201781. doi: 10.1016/j.omta.2026.201781 (PMC13355638; doi:10.1016/j.omta.2026.201781)
Supplement: Document S2. Article plus supplemental information [file mmc5.pdf]

Molecular Therapy
Advances
Original Article

Next generation AAV-F capsid gene therapy rescues disease pathology in a model of pyruvate dehydrogenase complex deficiency

Anna Keegan,[1] Özge Çetin,[1] Ellie M. Chilcott,[1] Juan Antinao Diaz,[1] Simon Eaton,[2] Simon N. Waddington,[1] John R. Counsell,[3] Shamima Rahman,[4] and Rajvinder Karda[1]

[1]EGA-Institute for Women's Health, University College London, London, UK; [2]Unit of Paediatric Surgery, UCL Institute of Child Health, London, UK; [3]Research Department of Targeted Intervention, UCL Division of Surgery and Interventional Science, Charles Bell House, London, UK; [4]Mitochondrial Research Group, Genetics and Genomic Medicine, UCL Great Ormond Street Institute of Child Health, and Metabolic Unit, Great Ormond Street Hospital for Children NHS Foundation Trust, London, UK

Pyruvate dehydrogenase complex deficiency (PDHD) is a severe mitochondrial disorder most frequently caused by pathogenic variants in *PDHA1*, leading to neurodevelopmental delay and early mortality, thus necessitating brain-targeted interventions. Using a brain-specific *Pdha1* knockout mouse model, we compared intracerebroventricular delivery of AAV9 capsid and a recently described synthetic neurotropic AAV-F capsid, both expressing human *PDHA1* coding sequence driven by a constitutive CAG promoter. Newborn mice received titer-matched AAV9, AAV-F, or AAV9 at 10-fold higher dose. Low-dose AAV-F and high-dose AAV9 significantly improved survival and restored PDH enzyme activity, metabolite profiles, and brain histopathology to near wild-type levels. However, mice treated by postnatal day 100 (P100) showed impaired motor function. Importantly, AAV-F achieved broad CNS transduction with minimal liver expression, thus outperforming low-dose AAV9. These results support the therapeutic potential of AAV-based gene therapy for PDHD and highlight AAV-F as a promising capsid for efficient, CNS-specific delivery.

INTRODUCTION

Pyruvate dehydrogenase complex (PDHc) deficiency is a metabolism disorder.[1] The PDHc enzyme complex converts pyruvate to acetyl-CoA, fueling the Krebs cycle and the subsequent generation of ATP via oxidative phosphorylation in the mitochondria.[2] A deficiency of PDHc leads to accumulation of pyruvate and lactate, decreased ATP production,[3] and disrupted neurotransmitter synthesis, particularly acetylcholine, glutamate, and γ-aminobutyric acid (GABA).[4] The resulting mitochondrial deficit particularly affects organs with high energy demands, such as the brain and the muscles,[5] and alters neuronal excitability and causes neurological symptoms.[6]

PDHc deficiency (PDHD) is inherited in an X-linked or autosomal recessive manner, with most cases caused by pathogenic variants in the X-chromosomal *PDHA1* gene, which encodes the E1-alpha subunit of PDHc.[7] Symptoms vary based on the severity of the specific genetic variant, residual enzyme activity,[8] and patient's sex. Hallmark features include central nervous system (CNS) abnormalities, developmental delay, hypotonia, ataxia, seizures,[4] encephalopathy, motor dysfunction, and severe lactic acidosis.[9] Neuroimaging often reveals ventriculomegaly and brain malformations such as hypogenesis of the corpus callosum.[10] Currently, a ketogenic diet is the recommended treatment and has been shown to improve motor function and reduce neuro-inflammation in patients with PDHD, but disease progression can continue.[11,12]

Adeno-associated virus serotype 9 (AAV9)-based gene therapies are being explored and applied in numerous preclinical and clinical trials for genetic disorders, particularly those affecting the CNS and neuromuscular system.[13–18] A key advantage of AAV9 is its ability to cross the blood-nervous system barrier, and its ability to deliver to the nervous system and visceral organs makes it useful for treating multi-system diseases such as neurometabolic diseases.[13] With high transduction of motor neurons, AAV9 has also shown great clinical efficacy in Zolgensma for spinal muscular atrophy via intravenous route.[19] High doses and volumes of AAV9 are required to achieve the desired therapeutic effects, but such high doses have been associated with off-target toxicities and immune responses.[20–22]

Synthetic modifications to naturally occurring capsids have allowed the development of novel capsids with greater transduction efficacy.[23] By the introduction of a ligand or peptide at a specific site on the capsid surface, potency and tropism are improved, leading to efficient targeting. These improvements can achieve a reduction in immunogenicity, as doses can be lowered without affecting the therapeutic effect. AAV-F, a novel synthetic AAV capsid derived

Received 13 August 2025; accepted 11 June 2026;
https://doi.org/10.1016/j.omta.2026.201781.

Correspondence: Rajvinder Karda, EGA Institute for Women's Health, University College London, London WC1E 6HX, UK.
E-mail: r.karda@ucl.ac.uk

Published by Elsevier Inc. on behalf of The American Society of Gene and Cell Therapy.
This is an open access article under the CC BY license (http://creativecommons.org/licenses/by/4.0/).

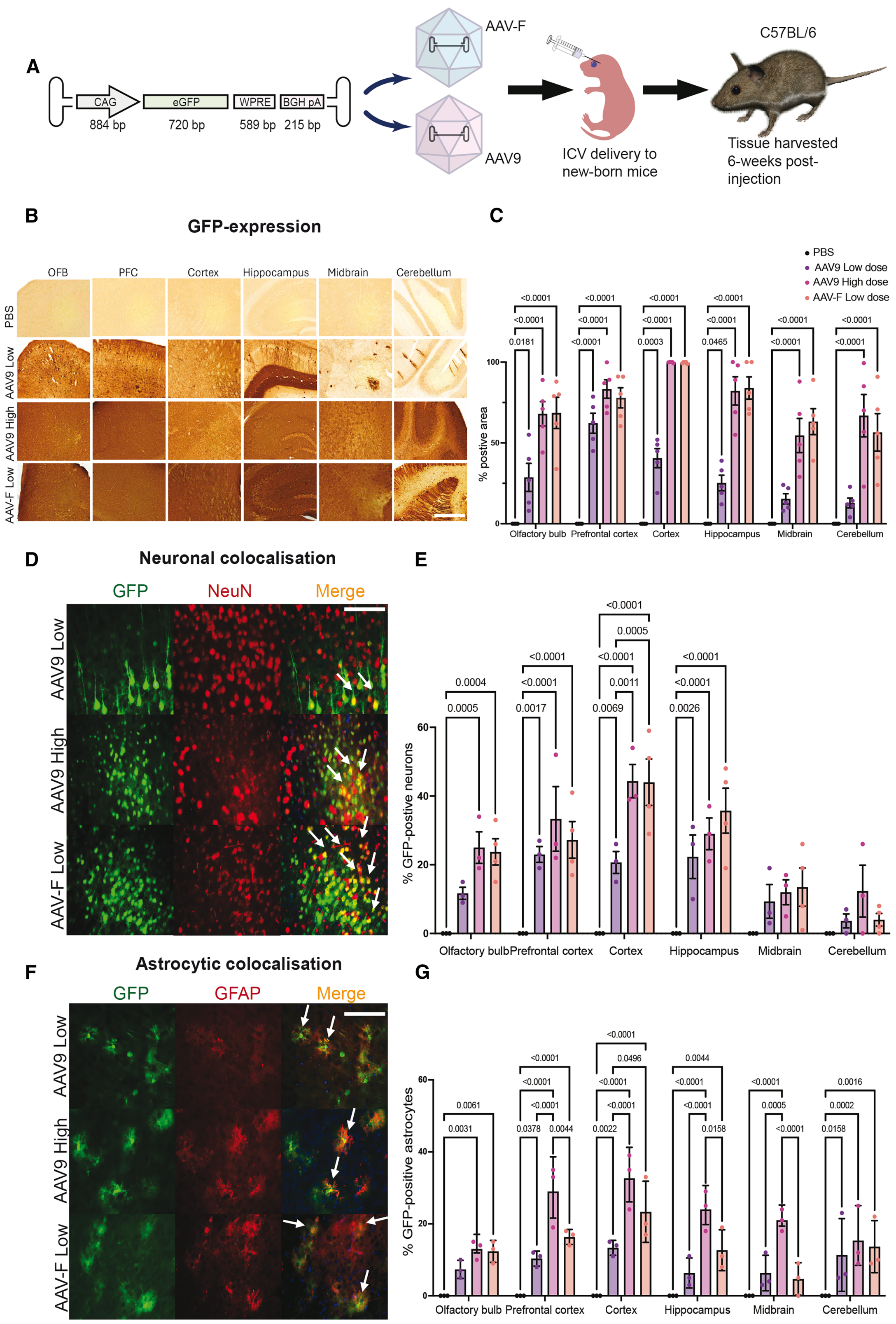

(legend on next page)

from AAV9, has been constructed with the aims of increasing tropism to the CNS and reducing the toxicity and immune responses associated with the parental capsid. AAV-F was generated by introducing a 7-amino acid insertion into the AAV9 Cap sequence. AAV-F demonstrated a greater CNS transduction profile after tail vein injections to adult mice.[24] AAV-F also showed greater transduction in human cortical neurons compared with AAV9.[24] When translated to non-human primates (NHPs), AAV-F had a greater vector copy number in spinal cord than titer-matched AAV9 after intrathecal delivery.[25] In addition, the AAV-F capsid was recently evaluated in a preclinical study to treat a mouse model of Dravet syndrome, where AAV-F-mediated therapy demonstrated superior therapeutic efficacy compared with AAV9.[26]

Previous studies have used an AAV2-CAG-*PDH-GFP* gene therapy vector and showed marked increase of PDHc enzymatic activity in patient fibroblast cells and positive GFP cells after stereotaxic injections to adult rats.[27] Self-complementary AAV2 (scAAV2) and scAAV6-CAG-*PDHA1-GFP* also showed increased E1α expression in patient fibroblasts.[28] However, neither of these approaches used a clinically relevant mouse model to test the therapeutic effects of AAV-mediated gene therapy.

Several animal models of PDHD have provided insights into disease mechanisms, particularly regarding metabolic dysregulation.[29,30] However, these existing models, especially systemic knockdowns of *Pdha1* exhibit embryonic lethality, limiting their utility for evaluating gene-replacement therapies.[29] A muscle-specific murine model has also been generated, but this phenotype does not reflect the predominantly neurological pathology observed in patients.[31,32] Therefore, a brain-specific model is required to more accurately capture disease-relevant features.

In this study, we utilized a brain-specific knockout (KO) mouse model of PDHD that closely recapitulates the human disease phenotype by reproducing the underlying patient neuropathology. The model was generated by the deletion of exon 8 of the X-chromosomal *Pdha1* gene,[5] and previous studies have demonstrated stunted growth, epilepsy, neuropathology (neuronal loss, increased glial fibrillary acidic protein [GFAP], cortical thinning, and hypoplasia of the corpus callosum), reduction of Pdha1 in neurons and astrocytes, and early death, specifically in male *Pdha1* KO mice.[5,33] In addition, we have shown that the model presents abnormal locomotion and motor defects. In this proof-of-concept study, we aimed to develop a one-off AAV-F-mediated gene supplementation therapy to deliver human *PDHA1* gene to the neonatal male *Pdha1* KO mice via unilateral intracerebroventricular (ICV) administration. We demonstrated that AAV-F gene therapy improved weight, survival, behavioral assessments, enzyme activity, metabolites, and neuropathology in treated *Pdha1* KO mice.

RESULTS

Widespread CNS gene expression after neonatal ICV administration of AAV-F to wild-type mice

To assess the expression profile of AAV-F after neonatal delivery, we administered AAV-F-CAG-*eGFP* (enhanced green fluorescence protein) at low dose (LD) (5×10^9 vg/pup) and AAV9-CAG-*eGFP* at LD and at high dose (HD) (5×10^{10} vg/pup) to newborn wild-type (WT) mice via unilateral ICV injections (Figure 1A). Five weeks later, we directly observed extensive GFP expression in the brain and visceral organs of AAV-F- and AAV9-treated groups (Figure S1A). Immunohistochemistry revealed comparable GFP expression throughout the brain with AAV-F-CAG-*eGFP* LD and AAV9-CAG-*eGFP* HD (Figure 1B). Quantification of immunoreactivity of GFP showed that AAV-F LD and AAV9 HD had comparable GFP expression in the prefrontal cortex, cortex, striatum, hippocampus, midbrain, and cerebellum (Figure 1C).

Co-localization studies were performed to determine neuronal and astrocyte targeting of AAV9 and AAV-F capsids in the brain after neonatal ICV administration. AAV-F-LD-treated mice demonstrated comparable GFP-positive neurons in the prefrontal cortex (27.25%), cortex (44%), and hippocampus (35.7%) to AAV9 HD (33.3%, 44.3%, and 29%, respectively) (Figures 1D, 1E, and S1B). AAV-F LD showed a significant higher percentage GFP-positive neurons in the cortex compared with titer-matched AAV9 (p = 0.0005) (Figure 1E). Astrocyte targeting was also observed with both AAV-F and AAV9 vectors (Figures 1F and S1C). Notably, AAV9 HD showed a significant astrocytic transduction in the prefrontal cortex (29%), hippocampus (18%), and midbrain (21%) when compared with AAV9 LD (10.3%, 6.3%, and 6.3%, respectively; $p < 0.0001$) and AAV-F LD (16.3%, $p = 0.0122$; 12.6%, $p = 0.0354$; and 4.6%; p = 0.0004; respectively) (Figure 1G). Overall, AAV-F LD showed comparable neuronal targeting in the brain compared to HD AAV9, whereas astrocytic targeting of AAV-F LD was comparable to AAV9 LD.

AAV-F-CAG-*hPDHA1* vector targets the brain with an acceptable safety profile

To assess the biodistribution and safety of the AAV-F-CAG-*hPDHA1* vector (Figure 2A), we administered AAV-F-CAG-*hPDHA1* LD and AAV9-CAG-*hPDHA1* LD and HD vectors to newborn WT mice via unilateral ICV injection. We observed no difference in the weight of treated groups compared with controls

Figure 1. AAV-F mediates a greater transduction efficiency of neurons and astrocytes than titer-matched AAV9 after neonatal ICV delivery
(A) Schematic diagram of neonatal biodistribution study. (B) Representative images of GFP expression in WT mice olfactory bulb, prefrontal cortex, cortex, hippocampus, midbrain, and cerebellum. (C) Stereological quantification of GFP-expression in WT mice. (D) Representative images of co-localization of NeuN and GFP in the cortex. Scale bars represent 100 μm. (E) Stereological quantification of NeuN and GFP immunofluorescence co-stain. One-way ANOVA, Tukey's multiple comparison ±SEM (n = 4). (F) Representative images of co-localization of GFAP and GFP in the cortex. Scale bars represent 100 μm. (G) Stereological quantification of GFAP and GFP immunofluorescence co-stain. One-way ANOVA, Tukey's multiple comparison ±SEM (n = 4).

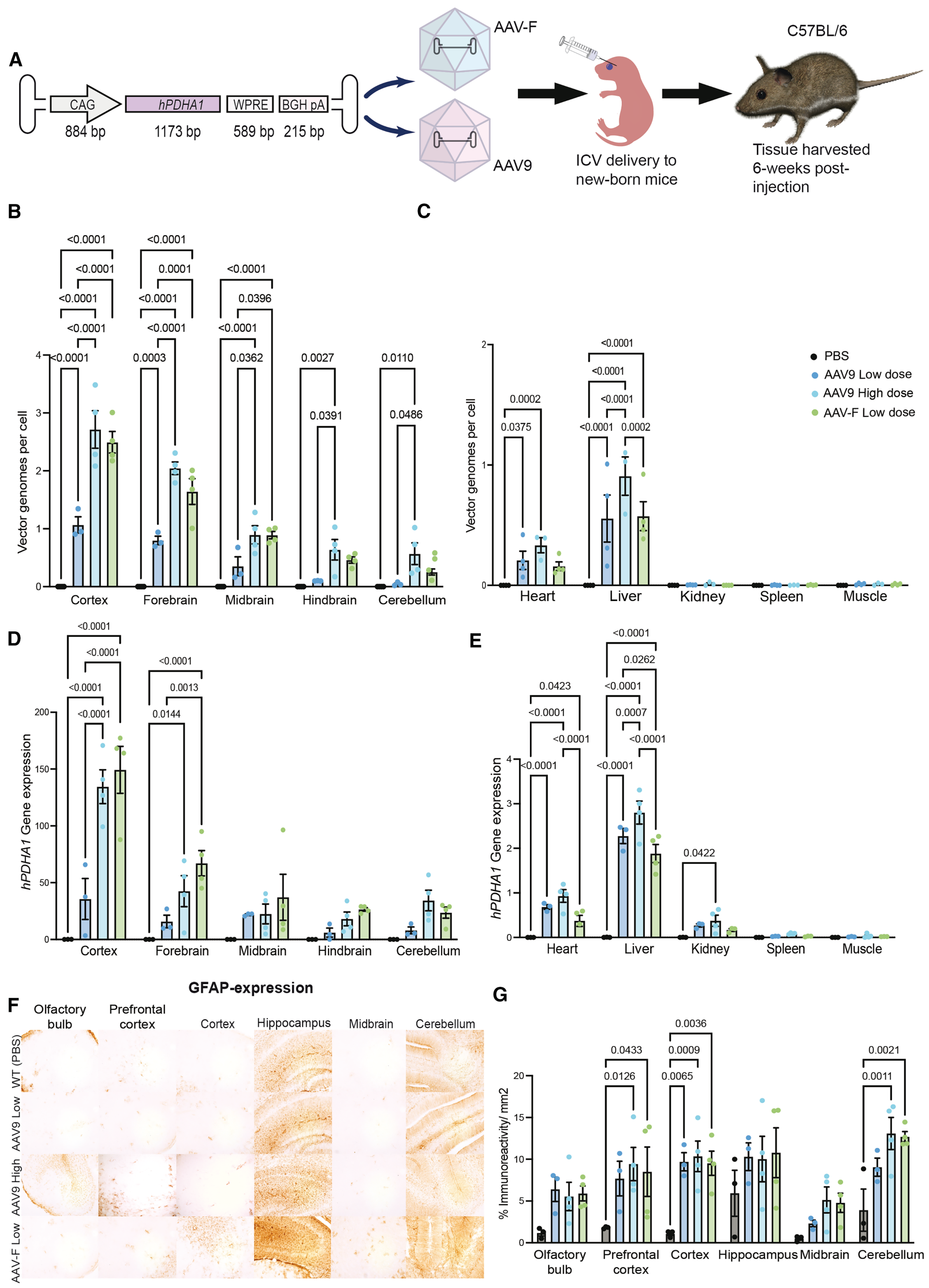

(legend on next page)

(Figure S2). Six weeks post-injection, vector genome copy number and *PDHA1* mRNA expression in the brain and visceral organs and neuropathology were assessed.

Vector genome copy number assessment was comparable for AAV9 HD and AAV-F LD groups throughout the brain (Figure 2B). AAV-F LD showed a significant increase in vector genome copy number in the cortex, forebrain, and midbrain compared with titer-matched AAV9 LD. Vector genome copy number analysis in the visceral organs showed significantly greater vector genome copy number in the liver of AAV9 HD group (0.9) compared with titer-matched AAV-F LD (0.57, p = 0.0002) and AAV9 LD (0.55, p < 0.0001) groups (Figure 2C). Comparable vector genome copy numbers were observed in the heart for all vectors and doses.

PDHA1 expression following AAV-F LD showed a significant increase in the cortex (p < 0.0001) and the forebrain (p = 0.0004) only when compared with those of titer-matched AAV9 group (Figure 2D). There was no significant difference throughout the brain between AAV-F LD and AAV9 HD groups (Figure 2D). In the visceral organs, AAV-F LD displayed no significant difference in *PDHA1* expression compared with titer-matched AAV9 (Figure 2E). AAV9 HD group had significantly higher *PDHA1* expression compared with AAV9 LD and AAV-F LD groups in the liver (AAV9 LD, p = 0.0007; AAV-F LD, p < 0.0001) (Figure 2E).

Immunohistochemistry for glial fibrillary acidic protein (GFAP; marker for astrocytes) and CD68 (marker for active microglia) demonstrated significant increase in GFAP expression in the prefrontal cortex (AAV9, p = 0.0126; AAV-F, p = 0.0433), cortex (AAV9, p = 0.0009; AAV-F, p = 0.0036), and cerebellum (AAV9, p = 0.0011; AAV-F, p = 0.0021) by AAV9 HD and AAV-F LD compared with PBS control group (Figures 2F and 2G). GFAP expression was significantly increased in AAV9 LD group in the cortex only (p = 0.0065) compared with PBS control group. There was no microglia activation in either any of the treated groups or PBS KO control group (Figure S3).

AAV-F-CAG-*hPDHA1* improved survival and neurological phenotype of *Pdha1* KO mice

Prior to testing the efficacy of our novel gene therapy, we first aimed to further characterize the phenotype of the *Pdha1* KO mouse model (Figures S4A–S4C). For the first time, we observed that this model presents with abnormal locomotion as measured by behavioral tests conducted at P16 of development, rotarod assessment, and open field activity (Figures S4D–S4G). There was a significant reduction in *mPdha1* expression at P20 of development, compared with age-matched WT controls (Figure S4H). Previous neuropathology assessment reported co-localized Pdha1 expression with GFAP and neurons (in the cortex, hippocampus, and cerebellum), cortical thinning, and hypoplasia of the corpus callosum.[5,33] Here, we observed widespread overexpression of GFAP and neuronal loss in the brain (Figures S4I and S4J).

Next, we sought to assess the efficacy of AAV-F-CAG-*hPDHA1* gene therapy treatment in male *Pdha1* KO mice. This was a blinded and randomized study. AAV-F-CAG-*hPDHA1* LD was administered via unilateral ICV injection by using AAV9-CAG-*hPDHA1* LD and HD as comparators (Figure 3A). PBS was delivered via unilateral ICV injection to *Pdha1* KO mice and WTs as control groups. We monitored the mice for 100 days of development. We observed 80% survival for AAV9 HD (p = 0.14), 75% for AAV-F LD (p = 0.1), 60% for AAV9 LD (p = 0.02), and 0% for PBS KO mice (p < 0.001), compared with 100% survival of PBS WT mice (Figure 3B). All groups had a significant improvement in survival compared with PBS KO mice (p < 0.0001). There was a significant difference in weight between all treatment groups compared with the control PBS KO group (p = 0.0005) (Figure 3C). There was no significant difference in weight between the mice in PBS WT group and the mice that received both AAV9 LD (p = 0.9409), AAV9 HD (p = 0.9235), and AAV-F LD (p = 0.9473) gene therapy at the end of the in-life experiment (Figure 3C).

Rotarod and open field behavioral assessments were performed at postnatal days (P) 16, P30, P50, and P100 to assess improvements in motor coordination and locomotion after AAV-F and AAV9 gene therapy. The rotarod assessment at P16 demonstrated that only the AAV-F LD (p = 0.0062) and PBS WT (p = 0.0028) groups had a significant improvement in time spent on the rotarod compared with the PBS KO group (Figures 3D and S5A). As the PBS KO mice died at P21/22 of development, the remaining behavioral time points were compared to PBS WT mice. Rotarod assessments at P30, P50, and P100 revealed significant differences in motor coordination in all AAV-F and AAV9 treatment groups compared with PBS-treated WT mice (Figure 3D). In contrast, open field assessment revealed a significant improvement at all doses for AAV-F and AAV9 treatment groups at P16 compared with PBS-treated KO mice (Figures 3E and S5B). Open field assessments at remaining time points showed comparable results between all AAV-F and AAV9 groups and the PBS WT control group (Figure 3E).

Figure 2. Neonatal intravenous delivery of AAV-F extensively transduces the CNS and visceral organs in WT mice

(A) Schematic diagram of therapeutic plasmid containing *hPDHA1.* (B) Vector genome analysis in discrete regions of the brain. One-way ANOVA, Tukey's multiple comparison ±SEM (n = 4). (C) Fold increase of *hPDHA1* to *mPdha1* (normalized to *mGapdh)* in discrete regions of the brain. One-way ANOVA, Tukey's multiple comparison ±SEM (n = 4). (D) Vector genome analysis of visceral organs. One-way ANOVA, Tukey's multiple comparison ±SEM (n = 4). (E) Fold increase of *hPDHA1* to *mPdha1* (normalized to *mGapdh)* in visceral organs. One-way ANOVA, Tukey's multiple comparison ±SEM (n = 4). (F) Representative images of GFAP expression in WT mice olfactory bulb, prefrontal cortex, cortex, hippocampus, midbrain and cerebellum. One-way ANOVA, with Dunnett's multiple comparison ±SEM. Images were taken at 400× magnification. Scale bars represent 100 μm. (G) Quantification (±SEM) of GFAP expression in the olfactory bulb (OFB), prefrontal cortex (PFC), cortex, striatum, hippocampus, midbrain, and cerebellum (n = 4).

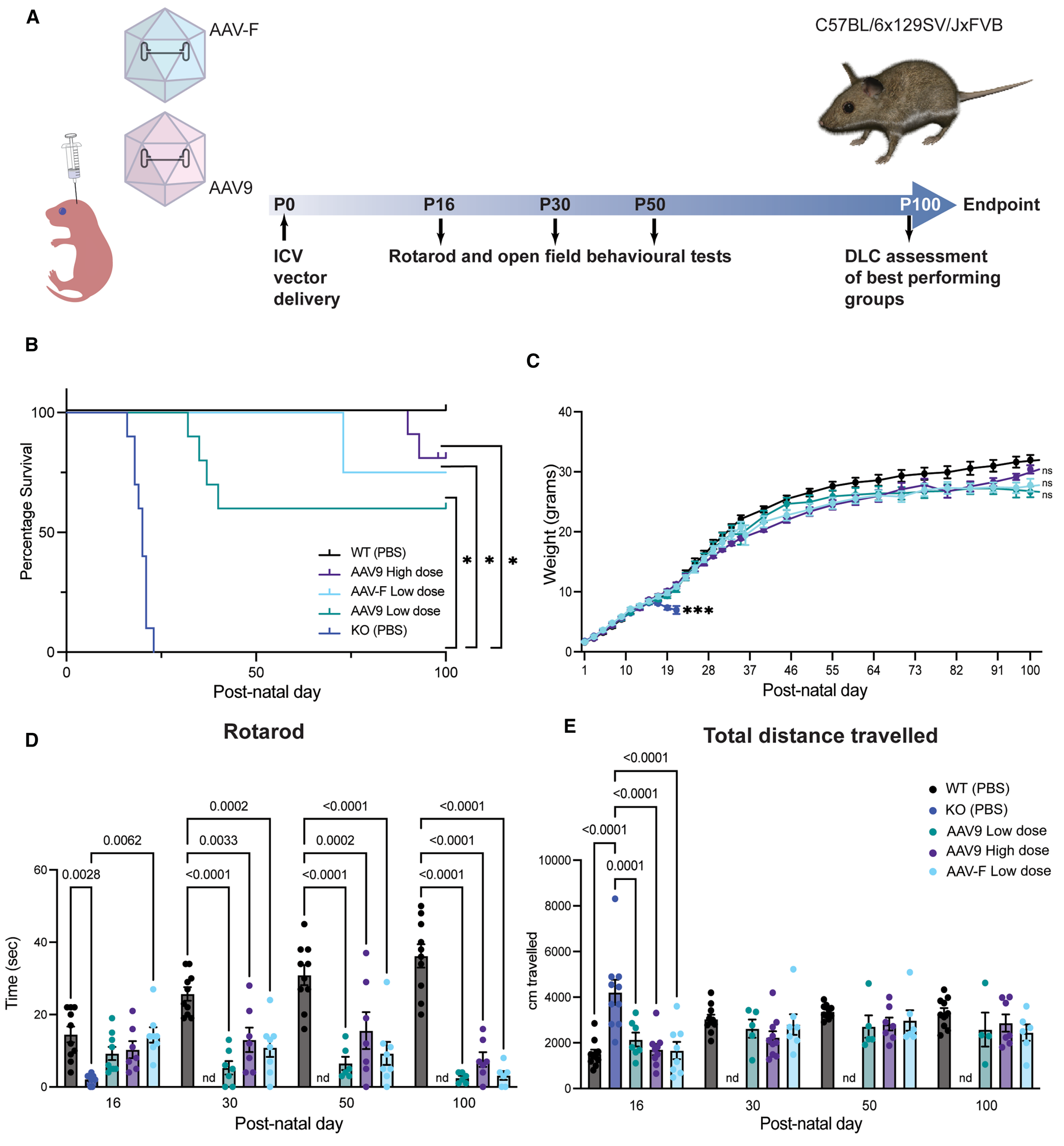

Figure 3. AAV-F gene therapy improves disease symptoms in a PDHD mouse model

(A) Schematic diagram illustrating the pre-clinical gene therapy study. (B) Kaplan-Meier survival curve. Data presented as a percentage of survival ($n = 8$ for AAV-F low dose, $n = 10$ for all other groups) (*$p < 0.0001$). (C) Weight shown as mean ± SEM (*$p < 0.0001$). Not significant, ns. (D) Behavioral rotarod assessment showing time spent on accelerated rotarod. One-way ANOVA, Tukey's multiple comparison ±SEM. (E) Open field test showing total distance traveled in 15 min. One-way ANOVA, Tukey's multiple comparison ±SEM. Not done, nd.

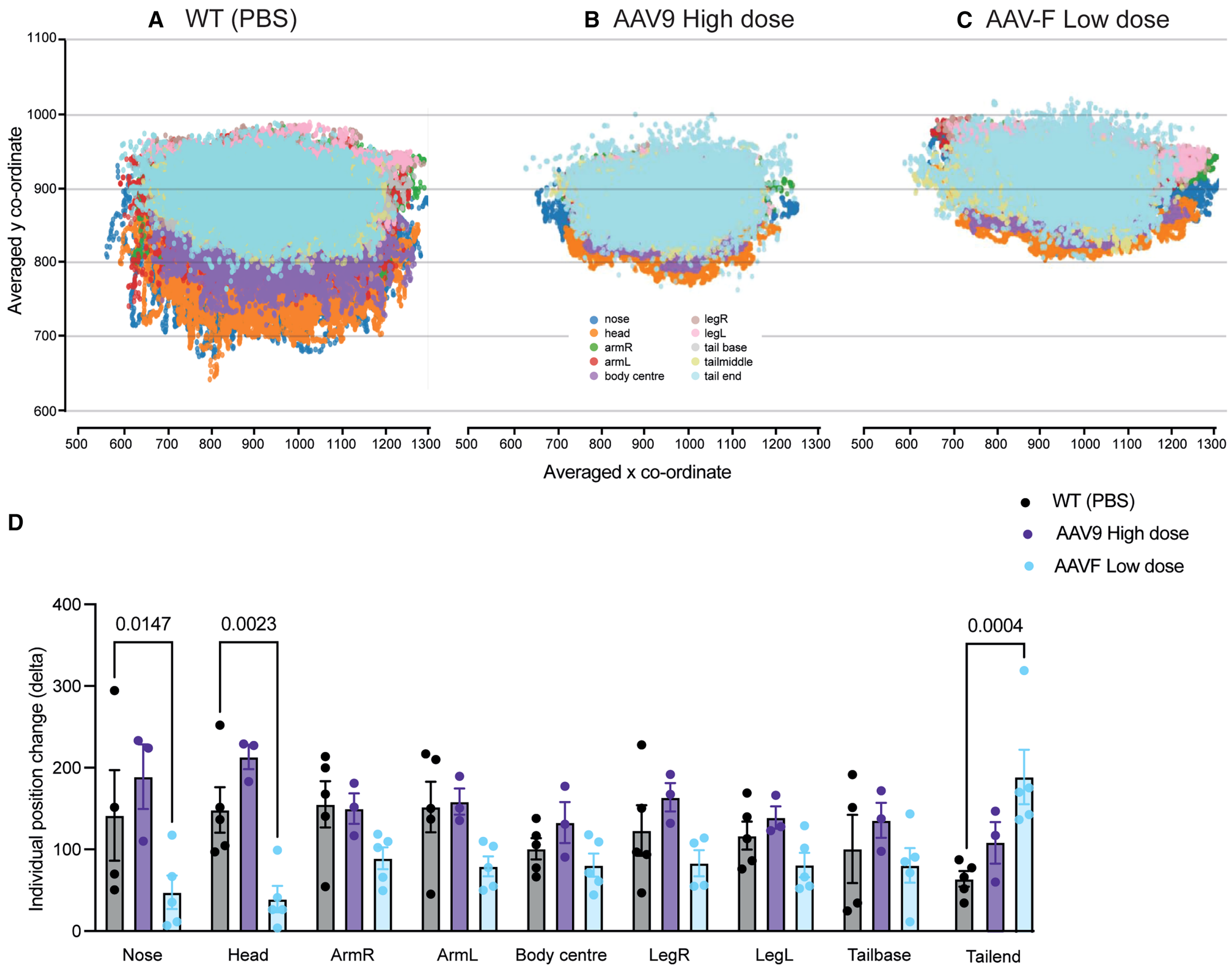

Figure 4. AI-behavioral assessment identifies abnormal phenotype

(A) Trajectory plot showing behavioral pose estimation of PBS-treated WT mice at postnatal day 100 (P100), recorded over a 15-min video period. Each body part is represented by a unique color: nose (blue), head (orange), right arm (green), left arm (red), body center (purple), right leg (dark purple), left leg (light pink), tail base (gray), tail middle (light green), and tail end (light blue). This color-coding applies consistently across all trajectory graphs. (B) Trajectory plot for AAV9 high-dose-treated KO mice. (C) Trajectory plots showing AAV-F low-dose-treated KO mice. (D) Quantification of body-part-specific movement based on *x-y* coordinates from Figure 4A–C. AAV-F low-dose-treated KO mice, showed a significant difference in the nose ($p = 0.0147$), head ($p = 0.0023$) and tailend ($p = 0.0004$) compared to PBS-treated WT mice. One-way ANOVA, Dunnett's multiple comparison.

As open field assessment failed to detect nuanced behavioral and motor abnormalities, novel AI assessment using DeepLabCut (DLC) was applied.[34] This behavioral assessment was used to quantify spontaneous locomotor activity and spatial movement patterns in adult mice using an objective, markerless tracking approach. By applying DLC-based pose estimation, the analysis enables unbiased measurement of body position and movement dynamics without the need for physical markers or experimenter scoring. The resulting positional and trajectory data provide sensitive metrics of overall motor function, exploratory behavior, and gross coordination, thus allowing the detection of subtle behavioral changes that may arise from this therapeutic intervention such as gait irregularities or impaired limb coordination. This approach allowed for a more sensitive and quantitative assessment of both disease-related motor deficits and treatment-induced improvements that were not apparent through conventional open field metrics (Figures 4A–4D and S6A–S6C).

This assessment was performed on mice treated with the best performing vector and dose, AAV-F LD and AAV9 HD, at P100. WT control mice showed more spread and variability, possibly suggesting more free movement (Figure 4A). The scatterplot for AAV9 high dose and AAV-F LD revealed a tighter cluster of distribution with constrained movement of head and tail. This indicated that the treated mice continued to exhibit an abnormal

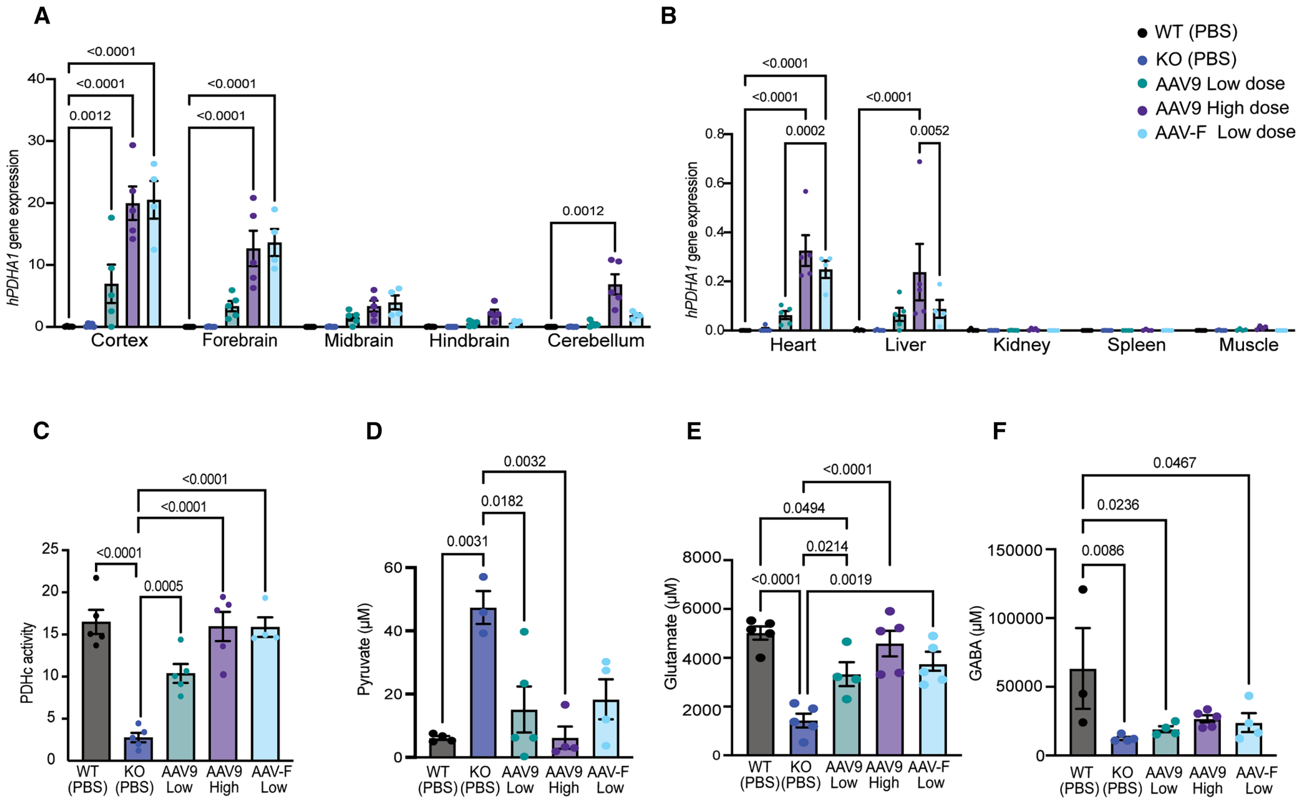

Figure 5. AAV-F low dose is significantly more efficient at restoring biochemical phenotype to WT levels than titer-matched AAV9

(A) Fold increase of *hPDHA1* to *mPdha1* (normalized to *mGapdh)* in discrete brain regions. One-way ANOVA, Tukey's multiple comparison ±SEM (*n* = 5). (B) Fold increase of *hPDHA1* to *mPdha1* (normalized to *mGapdh)* in visceral organs. One-way ANOVA, Tukey's multiple comparison ±SEM (*n* = 5). (C) PDHc enzyme activity in discrete regions of the brain. One-way ANOVA, Tukey's multiple comparison ±SEM (*n* = 5). (D) Pyruvate concentration in the brain. One-way ANOVA, Tukey's multiple comparison ±SEM (*n* = 5). (E) Glutamate concentration in the brain. One-way ANOVA, Tukey's multiple comparison ±SEM (*n* = 5). (F) GABA concentration in the brain. One-way ANOVA, Tukey's multiple comparison ±SEM (*n* = 5).

phenotype relative to age-matched WT controls (Figures 4B and 4C).

To quantify movement, we developed an algorithm to calculate total displacement based on *x* and *y* coordinates as shown in Figures 4A–4C (Figure S6). Figure 4D shows that while no significant differences were found for nose and head movements between the control and AAV9 HD groups, this was not sufficiently corrected with the AAV-F LD group. Tail displacement was notably higher in the AAV-F LD group than in the other groups (Videos S1, S2, and S3).

Long-term improvements in PDHc enzyme activity and metabolites in the brain following gene therapy

To assess the biodistribution of the vector, we examined *PDHA1* expression in discrete regions of the brain and visceral organs for all treatment groups. This was calculated based on fold-change of human *PDHA1* to mouse *Pdha1* (Figures S5C and S5D). We observed a significant increase in *PDHA1* expression in the cortex and forebrain following AAV9 HDHD and AAV-F LD ($p < 0.0001$) (Figure 5A). *PDHA1* expression was significantly higher in the liver of AAV9 HD group compared with AAV-F LD group ($p = 0.0052$). The heart revealed significant targeting with all vectors and doses (Figure 5B).

We observed significantly increased PDHc enzyme activity in AAV9 HD (15.95 mOD/min, $p < 0.0001$), AAV-F LD (15.88 mOD/min, $p < 0.0001$), and AAV9 LD (10.37 mOD/min, $p = 0.0005$) treatment groups compared with KO PBS-treated mice (2.5 mOD/min). AAV9 HD and AAV-F LD were comparable to PBS WT controls (16.29 moD/min) (Figure 5C).

To quantitatively assess metabolic alterations associated with disease progression and therapeutic intervention, gas chromatography was used to measure tissue concentrations of pyruvate, GABA, and glutamate. We assessed pyruvate concentrations in the brains of treated *Pdha1* KO mice as a biomarker for PDHD. Pyruvate levels were partially normalized to WT levels (6.1 µM) by AAV9 LD (15.1 µM), AAV9 HD (6.2 µM), and AAV-F LD (18.3 µM), while KO PBS-treated mice displayed 45.8 µM (Figure 5D).

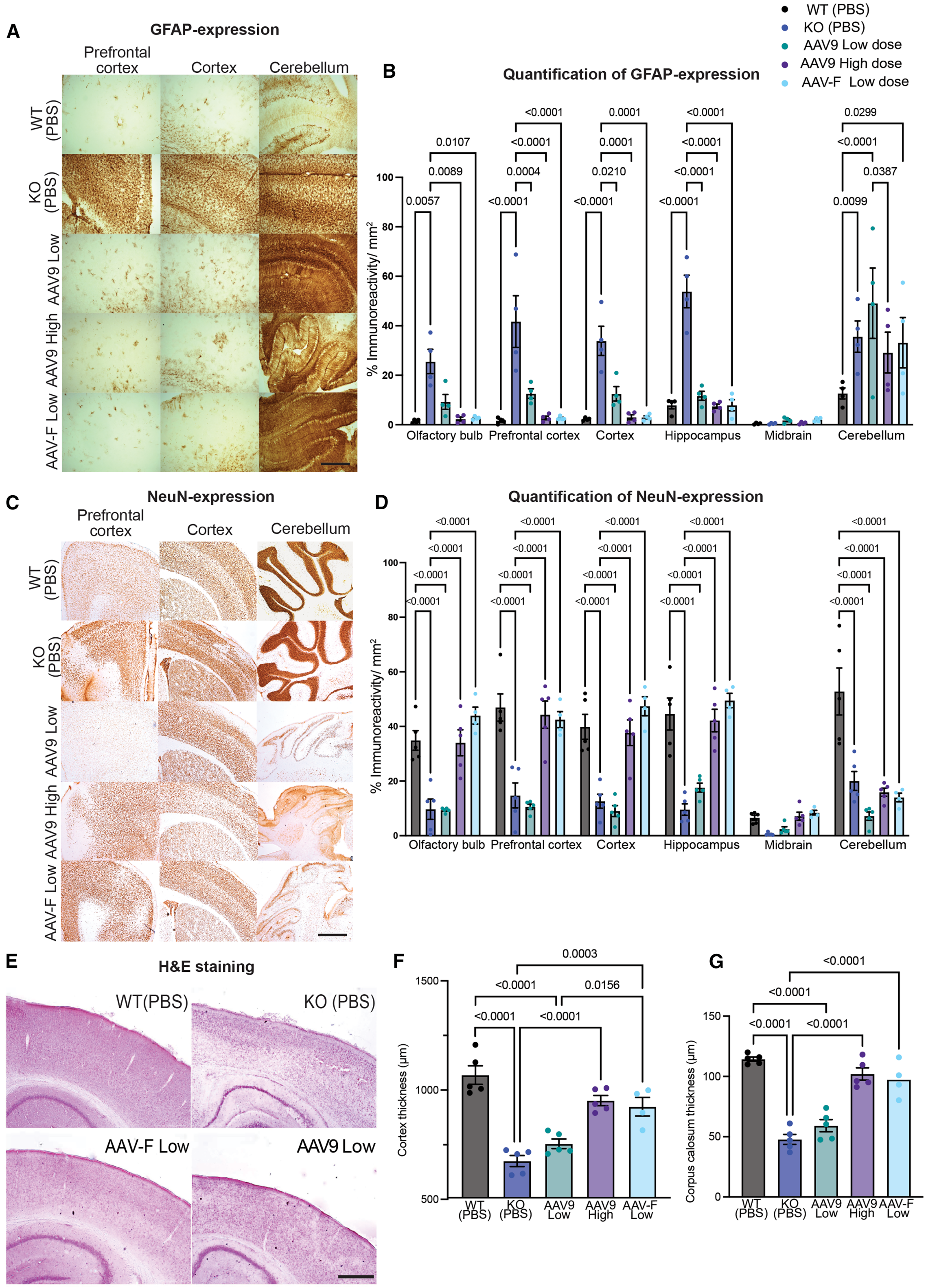

(legend on next page)

Levels of the neurotransmitters glutamate and GABA were quantified, since their concentrations are commonly altered as a result of PDH deficiency.[4] Glutamate levels were normalized in AAV9 HD (4582.64 μM) and AAV-F LD (3742.54 μM) groups, which were not significantly different from PBS WT (5015.02 μM) or PBS KO (1427.8 μM) groups (Figure 5E). AAV9 LD group ($p = 0.026$) showed a significantly lower concentration of glutamate compared with WT controls. GABA was partially rescued to PBS WT levels (63297 μM) by AAV9 HD (26678 μM) only (Figure 5F).

AAV-mediated gene therapy ameliorates neuropathology throughout the brain except for the cerebellum

To determine whether our novel AAV mediated gene therapy ameliorated the neuropathology in the *Pdha1* KO mice, we conducted GFAP, CD68, NeuN (marker for neurons), and hematoxylin and eosin (H&E) immunohistochemistry on brain tissue from AAV-F- and AAV9-treated mice. *Pdha1* KO mice suffer neuronal cell loss, upregulation of astrocytes and cortical/corpus callosum thinning, which, therefore, drives behavioral deficits seen in Figure 3.[33]

The results showed that there was a significant reduction in the upregulation of GFAP in AAV-F LD, AAV9 LD, and AAV9 HD groups throughout the brain except for the cerebellum, where titer-matched AAV-F LD ($p = 0.0011$) and AAV9 LD ($p < 0.0001$) displayed a significant elevation of GFAP-positive cells compared with WT controls (Figures 6A and 6B).

NeuN staining demonstrated a comparable neuronal immunoreactivity in the olfactory bulb, pre-frontal cortex, cortex, and hippocampus in AAV-F LD and AAV9 HD groups compared with PBS WT group. However, neuronal cell count was significantly reduced in the cerebellum for both AAV-F LD ($p < 0.001$) and AAV9 HD ($p < 0.001$) groups compared with PBS WT controls (Figures 6C and 6D). AAV9 LD showed significant reduction in neurons in the olfactory bulb, pre-frontal cortex, cortex, hippocampus, and cerebellum ($p < 0.0001$) (Figure 6D). CD68 stain showed no microglia activation throughout the brain in all treatment groups (Figure S7).

H&E staining was performed to measure cortical and corpus callosum thickness, as thinning of these are two biomarkers of neuropathology in both the *Pdha1* KO mice and patients with PDHD.[10] Cortical thinning was improved for both AAV-F LD and AAV9 HD groups (Figure 6E). Cortical thinning was significantly improved for both AAV-F LD ($p = 0.003$) and AAV9 HD ($p < 0.001$) groups compared with PBS-treated KO controls (Figure 6F). Cortical atrophy was not restored by AAV9 LD as there was no significant difference from PBS-treated KOs. In the corpus callosum, thinning was restored to WT levels by AAV-F LD and AAV9 HD treatment, whereas AAV9 LD-treated mice exhibited thinning similar to KO controls (Figure 6G). We demonstrated a significant improvement in cortical and corpus callosum thickening in AAV-F LD- and AAV9 HD-treated animals compared with PBS KO mice. Our findings suggest that our new therapy facilitates anatomical restoration and gives protection against neurodegeneration in PDHc-deficient mice.

DISCUSSION

This study demonstrated for the first time that our proof-of-concept AAV-F-mediated gene therapy can ameliorate the severe disease phenotype in a clinically relevant *Pdha1* KO mouse model, highlighting the potential of a one-off AAV-F gene therapy for PDHD.

AAV vectors, specifically AAV9, have been used extensively to treat neurological disorders, including spinal muscular atrophy.[19] However, high doses of AAV9 delivered via the i.v. route have been linked to liver toxicity and thrombotic microangiopathy.[22] Novel capsids that enable efficient therapeutic delivery at a lower dose are needed to avoid immunotoxicity associated with gene therapy. A novel AAV-F capsid has shown greater CNS transduction efficiency than titer-matched parental AAV9 capsid.[24,25]

In our proof-of-concept study, we have shown that AAV-F gene therapy to the brain of newborn PDHc-deficient mice at a lower dose than AAV9 (10-fold higher dose than AAV-F) can significantly extend life, reduce neurological disease symptoms, and restore PDHc enzyme activity and certain metabolites in the brain.

Prior to administering the *PDHA1* therapeutic vector to WT or *Pdha1* KO mice, we established the expression profile of AAV-F compared with titer-matched AAV9 expressing eGFP under the control of chicken beta-actin (CAG) promoter, after single unilateral ICV administration to WT neonatal mice. An additional dose of AAV9 at one log higher was also administered. This study revealed that at five weeks after a single neonatal ICV injection, the novel-engineered AAV-F capsid had enhanced neurotropism and CNS transduction rate compared with the parental AAV9 capsid. Previous studies have demonstrated significantly greater transduction of neurons and astrocytes in the CNS following adult tail vein administration of AAV-F compared with titer-matched AAV9, consistent with our findings.[24] The AAV-F capsid was engineered for enhanced CNS specificity and exhibits improved brain tropism relative to its parental AAV9 capsid. Importantly, AAV-F does not appear to

Figure 6. AAV-F low dose improves neuropathology in PDHc-deficient mice compared with titer-matched AAV9

(A) Representative images of GFAP expression in the prefrontal cortex (PFC), cortex, and cerebellum. (B) Quantification of GFAP expression in the olfactory bulb (OFB), PFC, cortex, hippocampus, midbrain, and cerebellum. One-way ANOVA, Tukey's multiple comparison ±SEM. (C) Representative images of neuronal loss in the PFC, cortex, and cerebellum. (D) Quantification of neuronal loss in the OFB, PFC, cortex, hippocampus, midbrain, and cerebellum. One-way ANOVA, Tukey's multiple comparison ±SEM. (E) Representative images of H&E staining of the cortex. (F) Quantification of cortex thickness. One-way ANOVA, Tukey's multiple comparison ±SEM. (G) Quantification of corpus callosum thickness. One-way ANOVA, Tukey's multiple comparison ±SEM.

rely on Ly6a for transduction; instead, recent work has shown that AAV-F interacts with Ly6c-family proteins, which likely contribute to its enhanced CNS tropism in murine models.[35] Accordingly, the improved transduction efficiency observed with AAV-F is attributable to capsid-receptor interactions distinct from Ly6a-dependent mechanisms.[36]

Although Ly6c-family receptor expression and function differ across species, studies in NHPs have demonstrated higher vector copy numbers of AAV-F in the spinal cord compared with titer-matched AAV9 following intrathecal delivery, thus supporting the translational potential of this capsid in larger animal models despite interspecies differences in receptor usage.[25] In this study, therapeutic efficacy was achieved using a low dose of 5×10^9 vector genomes per mouse delivered via ICV injection. Although direct dose extrapolation from mice to humans is not linear, ICV route of administration is already being used in clinical trials delivering AAV vectors to children with genetic neurological disorders (NCT05419492 and NCT06662188). Notably, the strong efficacy achieved at such low doses underscores the dose-sparing potential of this strategy, which supports improved safety margins and enhancing translational feasibility.[25]

We then administered AAV9 and AAV-F vectors expressing human *PDHA1* under the control of CAG promoter to WT and *Pdha1* KO mice by single unilateral ICV injection. The rationale behind the use of ICV injection was based on treating the brain as the main priority due to the severe neuropathology identified in previous studies.[33] In addition, the CNS is the primary affected area in patients and therefore, ICV injection would be the most applicable for clinical translation.[37] When the therapeutic vectors containing *PDHA1* were administered to WT mice, we observed no deaths and no statistical difference in weights compared with WT controls by the experimental endpoint. The vectors successfully delivered the *PDHA1* gene to the brain, with AAV-F showing higher vector genome copy number and more efficient gene expression, particularly in the cortex and midbrain. Interestingly, AAV-F at lower doses achieved similar expression to AAV9 at a higher dose, highlighting its potential for reduced vector dosing. While AAV9 HD had higher vector genome copy number in the cerebellum, this dose resulted in overexpression in the liver, which was significantly higher than untreated WT controls. AAV9 delivered at high dosages has broader tropism due to its ability to cross the blood brain barrier (BBB), its interaction with N-linked sialic acid receptors, and its systemic distribution.[38] Moreover, because the BBB remains naive during the neonatal period, the AAV vector has increased systemic targeting as it enters the bloodstream efficiently.[13] Liver targeting can be problematic in AAV-gene therapy via the i.v. route due to the risk of hepatotoxicity.[39] These data suggest that by delivering AAV-F via ICV route at 10-fold lower dose than AAV9, we can decrease liver targeting while maintaining high expression in the brain.

The *Pdha1* KO mouse model used in this gene therapy study was previously described by Jakkamsetti et al., with the *Pdha1* KO mice exhibiting stunted growth, epilepsy, neuropathology, and early death.[5] The *Pdha1* KO mice also showed a downregulation of PDHc enzyme activity and metabolite abnormalities (including glutamate and GABA), that closely recapitulate the human disease.[7,40] We further assessed this model and noted behavioral defects and neuropathology throughout the brain, including upregulation of astrocytes and neuronal loss.

In the gene therapy study, growth outcomes in treated mice were significantly improved compared with PBS-treated KO controls. AAV9 HD and AAV-F LD mice had the largest improvement in survival at 80% and 75%, respectively. Although 10-fold different in dose, there was no significant difference between either group's survival. AAV9 LD revealed a 60% survival which was significantly lower than titer-matched AAV-F LD. Behavioral assessment also demonstrated a dose-dependent improvement at P16. Initial rotarod assessment showed that the *Pdha1* KO mice spend less time on rotarod and have a lower latency to fall compared with WT mice. Rotarod assessment showed that AAV-F LD-treated mice spent longer on the rod than PBS-treated KO at P16. Although all treated groups had significantly lower latency to fall compared with WT controls at P30, P50, and P100, AAV9 HD had the longest time spent on the rod at all time points post-weaning. Open field studies showed similar results at P16, as there was no significant difference between the treated KOs and WT controls in total distance and mean speed. AAV9 LD and HD showed no significant decline in any of the open field assessments at P30, P50, or P100. AAV-F LD displayed a significantly lower mean speed at P100 compared with WT PBS controls. As the treated KO mice displayed an abnormal gait, which was identified in the rotarod assessment but undetected in the open field test, we aimed to identify another method that was more sensitive to assess the post-treatment phenotype. Therefore, an AI-based behavioral analysis tool (DLC) was used to assess whether the two most effective treatment groups (AAV-F LD and AAV9 HD) showed phenotypic improvements, as well as to identify additional behavioral changes not detected by standard open field testing. DLC analysis revealed that AAV-F treated *Pdha1* KO mice continued to exhibit abnormal locomotor phenotypes compared with PBS-treated WT controls. Specifically, there was a significant reduction in nose, head, and tail-end movement in the AAV-F treated group. Impaired tail dynamics, as measured by DLC, have previously been used to assess the severity of ataxia in mouse models of spinocerebellar ataxia type 6 (SCA6).[41] Tail movements are critical for balance in rodents, with active tail adjustments contributing significantly to postural stability, underscoring the tail's key role in locomotion.[42] Likewise, deficits in balance and gait ataxia are common features in various neurodegenerative disease models.[43,44] Additionally, abnormal head and nose movements have also been identified in prior DLC studies, supporting the sensitivity of this tool in detecting subtle motor impairments.[45,46] Future studies are required to compare AAV9 HD and AAV-F HD and assess whether increasing the dose of AAV-F can rectify the motor dysfunction observed in AAV-F LD group.

Brains of treated animals were assessed for gene expression, PDHc enzyme activity, Krebs cycle intermediates (pyruvate, GABA, and glutamate), and neuropathology. These metabolites were selected because they are known be disrupted in the *Pdha1* KO model.[5,33] The cortex and the forebrain had the highest *hPDHA1* expression with both doses of AAV9 and AAV-F LD vector, while AAV9 HD showed the greatest cerebellar targeting. Neither AAV vectors altered gene expression in the midbrain and hindbrain. As we used a brain-directed approach, lower visceral organ targeting was expected. Although AAV9 HD and AAV-F LD displayed similar results in the cortex and forebrain, *PDHA1* expression in the liver was significantly higher only in the AAV9 HD-treated group. Interestingly, AAV9 HD and AAV-F LD had a similar transduction in the heart. As there was little expression from AAV-F LD in the liver, it was not expected for the heart to show a significant increase. However, as these were neonatal ICV injections, the immature BBB has an increased permeability which allows an increase in cerebral blood flow.[47] The lack of rescue in visceral organs was not a priority of this study as visceral organ pathology is not a major clinical feature in human patients with PDHD.[32]

It was important to evaluate whether gene therapy could restore PDHc enzyme activity, pyruvate, alanine, glutamate, and GABA to WT levels.[5,33] We showed that AAV9 HD and AAV-F LD restored PDHc enzyme activity to that of WT PBS-treated mice. Our findings aligned with previous work, where a marked increase in PDHc enzyme activity was observed in patient fibroblasts cells with AAV2-CAG-*PDH-GFP*.[27] Future studies should involve western blot assessment to complement the PDHc enzyme activity assessed in the brain of treated mice.

AAV9 HD ameliorated the abnormalities in the selected metabolites as it decreased pyruvate and increased the neurotransmitters glutamate and GABA. AAV-F LD was also successful but not to the same extent, as GABA levels remained the same as the PBS KO group. Titer-matched AAV9 LD mice showed improvements in pyruvate, but glutamate and GABA concentrations were significantly lower than in PBS WT controls. AAV9 HD likely outperformed AAV-F LD in restoring neurotransmitter levels because it achieved higher and more widespread CNS transgene expression, particularly in critical neuronal subtypes responsible for glutamate and GABA synthesis. Future PDHc enzyme activity and metabolite assessment should be completed on discrete regions of the brain to indicate whether these disruptions are localized to the regions that lack therapeutic targeting.

The neuropathological analysis of the treated *Pdha1* KO mice confirmed that the neurological symptoms could be successfully ameliorated by AAV9 HD and AAV-F LD throughout the brain except for the cerebellum. Both groups reduced the upregulation of astrocytes, neuronal loss, cortical thinning, and hypoplasia of the corpus callosum. The observed improvements in cortical and corpus callosum thickness are likely a result of increased *PDHA1* expression in the cortex.

Additionally, tissues were collected at humane endpoints, which differed substantially between experimental groups due to treatment-dependent survival. PBS-treated KO mice were analyzed at an early humane endpoint at P20, whereas treated animals survived substantially longer and were analyzed at later humane endpoints, up to P100. Consequently, comparisons between PBS-treated KO and treated groups reflect different stages of disease progression rather than age-matched pathology. Importantly, the extended survival of treated animals is itself a key therapeutic outcome.

One limitation of this therapy was the lack of cerebellum targeting in all treatment groups. This results in an upregulation of GFAP astrocytes and reduced neuronal density. Despite ICV injections bypassing the BBB, the cerebellum sits below the fourth ventricle, and while the fourth ventricle is directly connected to the ventricular system, the cerebellum is further removed compared to other brain regions.[48,49] In addition, the cerebellum has a relatively different BBB compared with other brain regions and is not as well vascularized as the cerebral cortex, thus resulting in a reduction in vector delivery.[50] This absence of cerebellum targeting by this novel gene therapy requires further investigation; however, several pre-clinical studies have shown scarcity of expression in the cerebellum following ICV administration of AAV.[51,52] As there was no rescue of upregulation of astrocytes and neuronal death, disease progression continued as the mice advanced in age. Importantly, the lower neuronal counts observed in AAV-F LD- and AAV9 HD-treated animals were restricted to the cerebellum and perhaps reflect analysis at a later disease stage enabled by extended survival. In contrast, neuropathology in other brain regions was stabilized by treatment, indicating that cerebellar counts capture region-specific, survival-dependent progression rather than reduced overall therapeutic efficacy. Given the potential for dose-dependent effects, future comparative studies of high-dose AAV9 and AAV-F are needed to further assess the differences in efficacy, biodistribution, and safety. Our proof-of-concept study involved administration of AAV-mediated gene therapy to neonatal mice. Future studies will require testing our treatment in older mice which addresses a clinically relevant time point of administration. Treating >P15 mice is roughly equivalent to treating a child aged 1–2 years,[53] which is the current peak age of diagnosis of PDHc deficiency.[7]

Overall, this study provides compelling evidence that AAV-mediated gene supplementation therapy via ICV delivery rescues key disease pathological features in a PDHc-deficient mouse model. AAV9 HD and AAV-F LD significantly improved lifespan, abnormal brain architecture, and neurodegeneration. Importantly, the ability to achieve therapeutic benefit at a lower vector dose using AAV-F directly addresses a major barrier to clinical translation, namely the dose-dependent immunotoxicity and hepatotoxicity that have been observed with high-dose systemic AAV therapies.[54] The CNS-focused delivery strategy employed here is well aligned with the predominant neurological burden in PDHD patients, and ICV AAV delivery is being explored for pediatric neurogenetic disorders

(NCT05419492). Although neonatal administration represents an early intervention window, the observed stabilization of neuropathology and functional outcomes suggests that restoring PDHc activity during critical periods of brain development may prevent irreversible neurodegeneration. Collectively, these findings provide strong preclinical justification for further evaluation of AAV-F-mediated *PDHA1* gene therapy at later disease stages and in larger animals, with the goal of advancing a safe, one-off long-term, CNS-targeted gene therapy for children affected by PDHD.

MATERIALS AND METHODS

AAV production

AAV plasmid containing CAG promoter driving *eGFP* was acquired from Addgene (plasmid #37825). AAV plasmid encoding the human *PDHA1* gene driven by a CAG promoter was ordered from GENWIZ LTD (Germany).

The recombinant AAV vectors, AAV-F (Addgene plasmid # 166921) and AAV9 (University of Pennsylvania, PA, US), were generated using the triple plasmid transfection method described previously.[55] AKTA high-performance liquid chromatography (HPLC) with POROS CaptureSelect AAVX Affinity Resin (Thermo Fisher Scientific) virus purification was performed. Vectors were titered via Taqman quantitative polymerase chain reaction (qPCR) by QuantStudio (Thermo Fisher Scientific) and formulated in PBS.

Animal research

All animal studies were performed according to the Animal (Scientific Procedures) Act, 1986 and approved by the UK Home Office project license and ethical review board (AWERB). Animals were housed in a controlled environment with a 12-h light/dark cycle with food and water provided *ad libitum*. The biodistribution study was performed using WT C57BL/6J mice. The PDHc-deficient model that was used was previously described by Jakkamsetti et al.[5] by crossing a *Pdha1* flox female (B6.129P2-Pdha1tm1Ptl/J; Jackson Laboratory: 017443) with a *hGFAP*-Cre male (FVB-Tg(GFAP-cre)25Mes/J; 004600). Prior to this crossing, the *Pdha1* flox colony was moved onto a mixed C57BL/6J/129SV/J background.

All animal studies were blinded and randomized. ICV injections were performed on P0-P1 mice using a 33-gauge Hamilton needle, using coordinates described.[55] The biodistribution study used 5 μL of titer-matched AAV-F and AAV9 (5×10^9 vector genomes/pup). A third group was injected with AAV9 at a dose a log higher (5×10^{10} vector genomes/pup). The PDHc-deficient model was administered with 5 μL of titer-matched AAV-F and AAV9 containing the therapeutic gene (5×10^9 vector genomes/pup) (LD). The third group received a dose of 5×10^{10} vector genomes/pup (HD).

Animal behavioral analysis

All behavioral testing was done with the assessor blind to treatment and phenotype. Behavioral assessments were performed on P16, P30, P50, and P100. Three days prior to performing rotarod assessment, animals were trained to walk on the rotating rod. On the day of testing, the rotarod (Panlan LE8200, Cornella) was set to accelerate from 4 revolutions per minute (rpm) to 20 rpm. The trial ended when the animal fell off the rod. The time and speed at fall were noted and the trial was repeated two more times. Trials were re-run if the animal fell off the rotating rod within the first 5 s of the start time.

Harvard Panlab open field-testing equipment was used to assess the activity and general movements (Barcelona, Spain). A camera was suspended from the ceiling facing four 25 cm × 24 cm open field arenas. The animals were each placed in one of the arenas where they were allowed to move freely for 15 min. The movements were analyzed using SMART v.3 software (Harvard Panlab). Total distance traveled, time spent in periphery/center, total resting time, mean speed, and mean speed without resting were measured. The room was completely silent during recordings, with moderate lighting.

AI behavioral assessment

Mice were individually placed in a transparent Perspex arena (30 cm × 30 cm × 30 cm) and video-recorded for 15 min at P100. Behavior was recorded under controlled and consistent ambient lighting using a fixed side-view camera capturing 30 frames per second at a resolution of 1,920 × 1,080 pixels.

Video data were analyzed using DeepLabCut (DLC; v. 2.3.9), an open-source, markerless pose-estimation framework, implemented by following previously described protocols and the official GitHub repository (https://github.com/DeepLabCut).[34] To train the network, a total of 27,000 frames were manually annotated across representative videos. The following anatomically defined body parts were labeled: nose, head, right arm (armR), left arm (armL), body center, right leg (legR), left leg (legL), tail base, tail middle, and tail end. These annotated frames were used to train a convolutional neural network with a ResNet-50 backbone.

After iterative training and validation, the trained network was applied to all video frames to extract *x-y* coordinates for each labeled body part. Predictions below a predefined likelihood threshold were excluded to ensure tracking reliability. Movement was quantified separately for each tracked body part, thus enabling body-part-specific assessment of movement patterns rather than reliance on a single global locomotor metric.

For each body part, movement was calculated as frame-by-frame positional displacement ($\Delta\gamma = \Delta x, \Delta y$), and cumulative displacement over time was used as a quantitative measure of activity (supplemental methods). This approach allows detection of selective hyperactivity or hypoactivity in specific body regions even in the absence of large-scale changes in overall locomotion. Such physically derived kinematic measures provide an objective and observer-independent readout of abnormal movement patterns.

Animal tissue collection and processing

At the experimental endpoint, animals were placed under isoflurane anesthesia and perfused with phosphate-buffered saline (PBS, Sigma-Aldrich) via the left ventricle of the heart. The animal was then euthanized and relevant organs were collected. The left hemisphere of the brain was fixed following collection in 4% paraformaldehyde solution (Sigma-Aldrich) for 48 h before being moved to 30% sucrose for 24 h. Other harvested tissues were stored at −80°C until required. Tissue samples were collected at humane endpoints for all experimental groups. For treated animals, tissues were collected from mice that reached humane endpoints following therapeutic intervention, which occurred up to P100.

PBS-treated KO animals exhibited rapid disease progression and were euthanized at the humane endpoint at approximately P20. All tissues were processed using identical protocols by using all interim or non-endpoint samples.

qPCR analysis

DNA from tissue was extracted following the manufacturers' protocol for Qiagen DNeasy Blood & Tissue kit (Qiagen). Analysis of vector genome copy number per cell was determined using real-time quantitative PCR (qPCR) with Luna probe qPCR Master Mix (New England Biolabs). DNA was standardized so that every sample was 100 ng per µl. Absolute quantification was done using a standard curve for the housekeeping (m*Gapdh*) and target (*hPDHA1)* genes. Standards were prepared from gene fragments (Integrated DNA technologies). *hPDHA1* was normalized to the house keeping gene *mGapdh* to find vector genome copy number per diploid cell.

RNA was extracted using PureLink RNA purification kit (Thermofisher) and converted to cDNA with the Applied Biosystems high-capacity reverse transcription kit (Thermofisher). Absolute quantification was also used to determine the fold-change in gene expression per cell was determined using real-time qPCR with Luna probe qPCR Master Mix (New England Biolabs, UK). RNA was normalized so that every sample was 100 ng per µl prior to reverse transcription step. Primers were designed to target *mGapdh*, *mPdha1*, and *hPDHA1* (Table S1).

PDHc enzyme activity

PDHc enzyme activity in the brain was measured using the PDHc enzyme activity microplate assay kit from Abcam UK (ab109902). One half of a frozen brain hemisphere was homogenized in PBS to extract protein before determining sample protein concentration using the bicinchoninic acid (BCA) assay. 25 µL of detergent supplied in the kit to the 500 µL of sample and placed on ice for 10 min to solubilize. Following the incubation, the samples were centrifuged at 3,000 rpm for 10 min at 4°C. The supernatant was collected, and the samples were then diluted to 1 µg/µL in 400 µL using 1X buffer.

200 µL of sample was loaded into a well in the microplate, and the plate was covered and incubated at room temperature for 3 h. Following the 3-h incubation period, the wells were emptied and 300 µL of 1X stabilizer was added to each well. Absorbance of each sample was read at 450 nm every 20 s for 30 min. The enzyme activity was identified as:

$$\text{Rate (mOD / min)} = ((A2 - A1) \ / \ (T2 - T1)) / \ 0.001$$

Gas chromatography-mass spectrometry

The tissue collected was placed into a 15 mL conical tube where it received 5 mL of 5% acetic acid in methanol. The brain was homogenized at 20,000 rpm for 3 min using a mechanical homogenizer (IKA Labortechnick) and returned to an ice bath. The homogenates were centrifuged at 2,000 rpm for 30 min at 4°C. The supernatant was then transferred to a clean tube and processed immediately or returned to −80°C until required. Internal standards of glutamate, alpha-ketoglutarate, pyruvate, beta-hydroxybutyrate, and GABA were prepared as described previously. 100 mM solution of methoxylamine-hydrochloride (MOX) at pH 8–10 was prepared fresh daily by adding 0.0082 g of MOX to 10 mL of dH20. This solution was then diluted to 4 mM by adding 40–960 µL of dH20. 10 µL of the 4 mM MOX solution with 200 µL of sample and 10 µL of 1M internal standards were added to a glass vial, and the pH was adjusted with 10M NaOH to ensure a pH of 8–10. The vials were incubated for 3 h at 50°C on a heat block. After 3 h, the samples were transferred to a MAXI dry Iyo Heto Vacuum (MechaTech Systems, Bristol, UK) where they were vacuum-dried overnight. The next day, 100 µL of MSTFA (trifluoro-N-methyl-N-(trimethylsilyl)-acetamide) plus 1% TMCS (2,2,2-,chlorotrimethylsilane) (Sigma-Aldrich) was added to the vial, which was then incubated at 70°C for 50 min. Following the incubation, the samples were allowed to cool before transferring the 100 µL to a 1 mL gas vial. The sample was then injected into the gas chromatography-mass spectrometry (GC-MS) instrument. The machine used for GC-MS was ISQ 7610 Single Quadrupole (TRACE 1600) GC-MS (ThermoFisher Scientific). 2 µL sample was injected at 250°C at a split ratio of 1:10. Oven temperature started at 45°C and was increased to 200°C at 10°C per minute and then to 300°C at 30°C per minute. The MS was conducted at selected ion monitoring mode, monitoring for m/z 174 (pyruvate), 177 $^{13}C_3$ (pyruvate internal standard), 191 (lactate), 116 (alanine), 233 (beta-hydroxybutyrate), 237 $^{13}C_4$ (beta-hydroxybutyrate internal standard), 174 (GABA), 176 (d6-GABA internal standard, 246 (glutamate), 254 $^{13}C_5$ (glutamate internal standard). Standards of 500, 250, 100, 50 25, 10 and 0 µM were set up using the same process mentioned previously.

Immunofluorescence staining

Coronal 40 µm brain sections were cut with a microtome (Thermo Fisher Scientific). Sections were treated with 1% hydrogen peroxide for 30 min, then washed three times in 1× Tris-buffered saline (TBS). Non-specific binding sites were blocked with 15% goat serum (Vector Laboratories) solution in 0.3% Triton X-100 TBS (TBS-T) for 30 min. Representative sections were selected from each brain region under assessment. Immunolabeling was performed on floating

sections using antibodies directed against GFP (Abcam; ab290), NeuN (Merck; MAB377), and GFAP (Proteintech; 16825-1-AP) in 10% goat serum in TBS-T at 4°C overnight.

After 3 washes of 1× TBS, the sections were incubated in darkness with fluorescent secondary antibodies for 2 h; goat anti-chicken Alexa Fluor 488 (against GFP, Thermofisher scientific; A-11039), goat anti-mouse Alexa Fluor 568 (against NeuN, Thermofisher scientific; A-11004), and goat anti-chicken Alexa Fluor 568 (against GFAP, Thermofisher scientific; A-11041) in 10% goat serum in TBS-T at room temperature. Following 3 washes of 1× TBS, DAPI stain (4′,6-diamidino-2-phenylindole) (Thermofisher scientific) was added for 5 min, and the sections were mounted onto double-coated gelatinized slides. Once dried, a coverslip was added using Fluromount (Ebioscience). Image analysis was performed on a fluorescence microscope (Leica DFC7000 7) using LAS X Microscope Software (Leica Microsystems). Eight images were captured per region per mouse, and an average for each value was taken. The percentage GFP-positive neurons and astrocytes were analyzed using the CellProfiler Software (Broad Institute 2021). The images were overlaid using ImageJ.

Immunoperoxide staining

Coronal 40 μm brain sections were subjected to 1% hydrogen peroxide for 30 min, then washed three times in 1× TBS. Non-specific binding sites were blocked with 15% goat serum (Vector Laboratories) solution in 0.3% Triton X-100 TBS (TBS-T) for 30 min. Immunolabeling was performed on floating sections using antibodies directed against GFP (Abcam), 10% goat serum in TBS-T at 4°C overnight. This protocol has been described by Karda et al.[56]

H&E staining

Coronal 40 μm brain sections were subjected to H&E staining with 4 sections taken with reference to position S71 of the Allen Brain Atlas Map, 2024 (http://mouse.brain-map.org.) where the hippocampus has not yet turned. Brains were prepared and stained as per the protocol previously described Massaro et al.[57]

Cortex and corpus callosum measurement

ImageJ was used to measure the distance from the outer cortical plate to the ventricular zone (Allen Brain Atlas Map). The corpus callosum was also measured from position S71. An average of four measurements was taken.

Statistics

Statistical analysis was performed on GraphPad Prism v. 9.3.1, GraphPad Software, San Diego, California, USA (www.graphpad.com). *p* values that were less than 0.05 were taken to be statistically significant. Survival analysis was completed using the Mantel-Cox test. Weights were taken every other day and analyzed with repeated measures ANOVA. Two-way ANOVA with Tukey's multiple comparison tests was used for GFP-positive neurons and astrocytes, vector genome analysis, rotarod, open field, qPCR, PDHc enzyme activity, and neuropathology assessments. Data are presented as mean and standard error of the mean (SEM).

DATA AND CODE AVAILABILITY

All data associated with this study are present in the paper in supplemental information. Requests for data should be addressed to R.K.

ACKNOWLEDGMENTS

This work was funded by LifeArc (P2020-0008 and P2023-0011 to R.K., J.A.D., E.M.C, and S.N.W.); Great Ormond Street Hospital Children's Charity, UK and Dravet Syndrome UK Charity (V4720 and V4919 to R.K., J.A.D., S.N.W., and E.M.C.); Therapeutic Acceleration Support (TAS), UCL (to R.K., A.K., and S.R);. Medical Research Council Development Pathway Funding Scheme, UK (MR/Z505201/1 to R.K. and E.M.C). and Freya Foundation Charity, UK.

AUTHOR CONTRIBUTIONS

R.K., S.R., A.K., and J.R.C, designed the research; R.K. and A.K. drafted the manuscript; A.K., Ö.Ç., J.A.D., and E.M.C., performed the experimental studies; A.K., and Ö.Ç., analyzed data; R.K., S.R., S.N.W., S.E., and A.K. guided research. All authors agreed to the final version of this manuscript.

DECLARATION OF INTERESTS

The authors declare no competing interests.

SUPPLEMENTAL INFORMATION

Supplemental information can be found online at https://doi.org/10.1016/j.omta.2026.201781.

REFERENCES

1. Ferreira, C.R., Rahman, S., Keller, M., Zschocke, J., ICIMD Advisory Group, Ali, H., Artuch, R., Ballabio, A., Barshop, B., Baumgartner, M., et al. (2021). An international classification of inherited metabolic disorders (ICIMD). J. Inherit. Metab. Dis. *44*, 164–177. https://doi.org/10.1002/jimd.12348.
2. Zangari, J., Petrelli, F., Maillot, B., and Martinou, J.C. (2020). The multifaceted pyruvate metabolism: Role of the mitochondrial pyruvate carrier. Biomolecules *10*, 1068. https://doi.org/10.3390/biom10071068.
3. Lissens, W., De Meirleir, L., Seneca, S., Liebaers, I., Brown, G.K., Brown, R.M., Ito, M., Naito, E., Kuroda, Y., Kerr, D.S., et al. (2000). Mutations in the X-linked pyruvate dehydrogenase (E1) α subunit gene (PDHA1) in patients with a pyruvate dehydrogenase complex deficiency. Hum. Mutat. *15*, 209–219. https://doi.org/10.1002/(SICI)1098-1004(200003)15:3<209::AID-HUMU1>3.0.CO;2-K.
4. Bhandary, S., and Aguan, K. (2015). Pyruvate dehydrogenase complex deficiency and its relationship with epilepsy frequency - An overview. Epilepsy Res. *116*, 40–52. https://doi.org/10.1016/j.eplepsyres.2015.07.002.
5. Jakkamsetti, V., Marin-Valencia, I., Ma, Q., Good, L.B., Terrill, T., Rajasekaran, K., Pichumani, K., Khemtong, C., Hooshyar, M.A., Sundarrajan, C., et al. (2019). Brain metabolism modulates neuronal excitability in a mouse model of pyruvate dehydrogenase deficiency. Sci. Transl. Med. *11*, eaan0457. https://doi.org/10.1126/scitranslmed.aan0457.
6. Barnerias, C., Saudubray, J.M., Touati, G., De Lonlay, P., Dulac, O., Ponsot, G., Marsac, C., Brivet, M., and Desguerre, I. (2010). Pyruvate dehydrogenase complex deficiency: Four neurological phenotypes with differing pathogenesis. Dev. Med. Child Neurol. *52*, e1–e9. https://doi.org/10.1111/j.1469-8749.2009.03541.x.
7. DeBrosse, S.D., Okajima, K., Zhang, S., Nakouzi, G., Schmotzer, C.L., Lusk-Kopp, M., Frohnapfel, M.B., Grahame, G., and Kerr, D.S. (2012). Spectrum of neurological and survival outcomes in pyruvate dehydrogenase complex (PDC) deficiency: Lack of correlation with genotype. Mol. Genet. Metab. *107*, 394–402. https://doi.org/10.1016/j.ymgme.2012.09.001.
8. Head, R.A., Brown, R.M., Zolkipli, Z., Shahdadpuri, R., King, M.D., Clayton, P.T., and Brown, G.K. (2005). Clinical and genetic spectrum of pyruvate dehydrogenase deficiency: Dihydrolipoamide acetyltransferase (E2) deficiency. Ann. Neurol. *58*, 234–241. https://doi.org/10.1002/ana.20550.

9. Keshavan, N., and Rahman, S. (2018). Natural history of mitochondrial disorders: A systematic review. Essays Biochem. *62*, 423–442. https://doi.org/10.1042/EBC20170108.

10. Savvidou, A., Ivarsson, L., Naess, K., Eklund, E.A., Lundgren, J., Dahlin, M., Frithiof, D., Sofou, K., and Darin, N. (2022). Novel imaging findings in pyruvate dehydrogenase complex (PDHc) deficiency—Results from a nationwide population-based study. J. Inherit. Metab. Dis. *45*, 248–263. https://doi.org/10.1002/jimd.12463.

11. Branco, A.F., Ferreira, A., Simões, R.F., Magalhães-Novais, S., Zehowski, C., Cope, E., Silva, A.M., Pereira, D., Sardão, V.A., and Cunha-Oliveira, T. (2016). Ketogenic diets: From cancer to mitochondrial diseases and beyond. Eur. J. Clin. Invest. *46*, 285–298. https://doi.org/10.1111/eci.12591.

12. Ułamek-Kozioł, M., Czuczwar, S.J., Pluta, R., and Januszewski, S. (2019). Ketogenic diet and epilepsy. Nutrients *11*, 2510. https://doi.org/10.3390/nu11102510.

13. Foust, K.D., Nurre, E., Montgomery, C.L., Hernandez, A., Chan, C.M., and Kaspar, B.K. (2009). Intravascular AAV9 preferentially targets neonatal neurons and adult astrocytes. Nat. Biotechnol. *27*, 59–65. https://doi.org/10.1038/nbt.1515.

14. Gray, S.J., Matagne, V., Bachaboina, L., Yadav, S., Ojeda, S.R., and Samulski, R.J. (2011). Preclinical differences of intravascular aav9 delivery to neurons and glia: A comparative study of adult mice and nonhuman primates. Mol. Ther. *19*, 1058–1069. https://doi.org/10.1038/mt.2011.72.

15. Di Meo, I., Marchet, S., Lamperti, C., Zeviani, M., and Viscomi, C. (2017). AAV9-based gene therapy partially ameliorates the clinical phenotype of a mouse model of Leigh syndrome. Gene Ther. *24*, 661–667. https://doi.org/10.1038/gt.2017.53.

16. Bey, K., Ciron, C., Dubreil, L., Deniaud, J., Ledevin, M., Cristini, J., Blouin, V., Aubourg, P., and Colle, M.A. (2017). Efficient CNS targeting in adult mice by intrathecal infusion of single-stranded AAV9-GFP for gene therapy of neurological disorders. Gene Ther. *24*, 325–332. https://doi.org/10.1038/gt.2017.18.

17. Meyer, K., Ferraiuolo, L., Schmelzer, L., Braun, L., McGovern, V., Likhite, S., Michels, O., Govoni, A., Fitzgerald, J., Morales, P., et al. (2015). Improving single injection CSF delivery of AAV9-mediated gene therapy for SMA: A dose-response study in mice and nonhuman primates. Mol. Ther. *23*, 477–487. https://doi.org/10.1038/mt.2014.210.

18. Guo, W., Rioux, M., Shaffo, F., Hu, Y., Yu, Z., Xing, C., and Gray, S.J. (2024). AAV9/SLC6A1 gene therapy rescues abnormal EEG patterns and cognitive behavioral deficiencies in Slc6a1-/- mice. J. Clin. Investig. *135*, e182235. https://doi.org/10.1172/JCI182235.

19. Strauss, K.A., Swoboda, K.J., Farrar, M.A., McMillan, H.J., Parsons, J., Krueger, J.M., Iannaccone, S.T., Chiriboga, C.A., Kwon, J.M., Saito, K., et al. (2019). Onasemnogene abeparvovec gene-replacement therapy (GRT) in presymptomatic spinal muscular atrophy (SMA): SPR1NT study update. J. Neurol. Sci. *405*, 268–269. https://doi.org/10.1016/j.jns.2019.10.1317.

20. Hinderer, C., Katz, N., Buza, E.L., Dyer, C., Goode, T., Bell, P., Richman, L.K., and Wilson, J.M. (2018). Severe Toxicity in Nonhuman Primates and Piglets Following High-Dose Intravenous Administration of an Adeno-Associated Virus Vector Expressing Human SMN. Hum. Gene Ther. *29*, 285–298. https://doi.org/10.1089/hum.2018.015.

21. Hordeaux, J., Hinderer, C., Goode, T., Katz, N., Buza, E.L., Bell, P., Calcedo, R., Richman, L.K., and Wilson, J.M. (2018). Toxicology Study of Intra-Cisterna Magna Adeno-Associated Virus 9 Expressing Human Alpha-L-Iduronidase in Rhesus Macaques. Mol. Ther. Methods Clin. Dev. *10*, 79–88. https://doi.org/10.1016/j.omtm.2018.06.003.

22. Chand, D., Mohr, F., McMillan, H., Tukov, F.F., Montgomery, K., Kleyn, A., Sun, R., Tauscher-Wisniewski, S., Kaufmann, P., and Kullak-Ublick, G. (2021). Hepatotoxicity following administration of onasemnogene abeparvovec (AVXS-101) for the treatment of spinal muscular atrophy. J. Hepatol. *74*, 560–566. https://doi.org/10.1016/j.jhep.2020.11.001.

23. Büning, H., and Srivastava, A. (2019). Capsid Modifications for Targeting and Improving the Efficacy of AAV Vectors. Mol. Ther. Methods Clin. Dev. *12*, 248–265. https://doi.org/10.1016/j.omtm.2019.01.008.

24. Hanlon, K.S., Meltzer, J.C., Buzhdygan, T., Cheng, M.J., Sena-Esteves, M., Bennett, R.E., Sullivan, T.P., Razmpour, R., Gong, Y., Ng, C., et al. (2019). Selection of an Efficient AAV Vector for Robust CNS Transgene Expression. Mol. Ther. Methods Clin. Dev. *15*, 320–332. https://doi.org/10.1016/j.omtm.2019.10.007.

25. Beharry, A., Gong, Y., Kim, J.C., Hanlon, K.S., Nammour, J., Hieber, K., Eichler, F., Cheng, M., Stemmer-Rachamimov, A., Stankovic, K.M., et al. (2022). The AAV9 Variant Capsid AAV-F Mediates Widespread Transgene Expression in Nonhuman Primate Spinal Cord after Intrathecal Administration. Hum. Gene Ther. *33*, 61–75. https://doi.org/10.1089/hum.2021.069.

26. Chilcott, E.M., Diaz, J.F.A., Barbanoj, A.A., Moore, M., Keegan, A., Waddington, Z., Fang, V., Schorge, S., Lignani, G., Waddington, S.N., and Karda, R. (2025). AAV delivery of RNA editing machinery rescues SUDEP and seizure phenotype in a mouse model of Dravet Syndrome. Preprint at bioRxiv. https://doi.org/10.1101/2025.07.18.664895.

27. Owen, R., 4th, Mandel, R.J., Ammini, C.V., Conlon, T.J., Kerr, D.S., Stacpoole, P.W., and Flotte, T.R. (2002). Gene therapy for pyruvate dehydrogenase E1α deficiency using recombinant adeno-associated virus 2 (rAAV2) vectors. Mol. Ther. *6*, 394–399. https://doi.org/10.1006/mthe.2002.0683.

28. Han, Z., Berendzen, K., Zhong, L., Surolia, I., Chouthai, N., Zhao, W., Maina, N., Srivastava, A., and Stacpoole, P.W. (2008). A combined therapeutic approach for pyruvate dehydrogenase deficiency using self-complementary adeno-associated virus serotype-specific vectors and dichloroacetate. Mol. Genet. Metab. *93*, 381–387. https://doi.org/10.1016/j.ymgme.2007.10.131.

29. Johnson, M.T., Mahmood, S., Hyatt, S.L., Yang, H.S., Soloway, P.D., Hanson, R.W., and Patel, M.S. (2001). Inactivation of the murine pyruvate dehydrogenase (Pdha1) gene and its effect on early embryonic development. Mol. Genet. Metab. *74*, 293–302. https://doi.org/10.1006/mgme.2001.3249.

30. Taylor, M.R., Hurley, J.B., Van Epps, H.A., and Brockerhoff, S.E. (2004). A zebrafish model for pyruvate dehydrogenase deficiency: Rescue of neurological dysfunction and embryonic lethality using a ketogenic diet. Proc. Natl. Acad. Sci. USA *101*, 4584–4589. https://doi.org/10.1073/pnas.0307074101.

31. Gopal, K., Abdualkader, A.M., Li, X., Greenwell, A.A., Karwi, Q.G., Altamimi, T.R., Saed, C., Uddin, G.M., Darwesh, A.M., Jamieson, K.L., et al. (2023). Loss of muscle PDH induces lactic acidosis and adaptive anaplerotic compensation via pyruvate-alanine cycling and glutaminolysis. J. Biol. Chem. *299*, 105375. https://doi.org/10.1016/j.jbc.2023.105375.

32. Patel, K.P., O'Brien, T.W., Subramony, S.H., Shuster, J., and Stacpoole, P.W. (2012). The spectrum of pyruvate dehydrogenase complex deficiency: Clinical, biochemical and genetic features in 371 patients. Mol Genet Metab *106*, 385–394. https://doi.org/10.1016/j.ymgme.2012.03.017.

33. Marin-Valencia, I., Kocabas, A., Rodriguez-Navas, C., Miloushev, V.Z., González-Rodríguez, M., Lees, H., Henry, K.E., Vaynshteyn, J., Longo, V., Deh, K., et al. (2024). Imaging brain glucose metabolism in vivo reveals propionate as a major anaplerotic substrate in pyruvate dehydrogenase deficiency. Cell Metab. *36*, 1394–1410.e12. https://doi.org/10.1016/j.cmet.2024.05.002.

34. Mathis, A., Mamidanna, P., Cury, K.M., Abe, T., Murthy, V.N., Mathis, M.W., and Bethge, M. (2018). DeepLabCut: markerless pose estimation of user-defined body parts with deep learning. Nat. Neurosci. *21*. https://doi.org/10.1038/s41593-018-0209-y.

35. Huang, Q., Chan, K.Y., Tobey, I.G., Chan, Y.A., Poterba, T., Boutros, C.L., Balazs, A.B., Daneman, R., Bloom, J.M., Seed, C., and Deverman, B.E. (2019). Delivering genes across the blood-brain barrier: LY6A, a novel cellular receptor for AAV-PHP.B capsids. PLoS One *14*, e0225206. https://doi.org/10.1371/journal.pone.0225206.

36. Huang, Q., Chen, A.T., Chan, K.Y., Sorensen, H., Barry, A.J., Azari, B., Zheng, Q., Beddow, T., Zhao, B., Tobey, I.G., et al. (2023). Targeting AAV vectors to the central nervous system by engineering capsid–receptor interactions that enable crossing of the blood–brain barrier. PLoS Biol. *21*, e3002112. https://doi.org/10.1371/journal.pbio.3002112.

37. Massaro, G., Geard, A.F., Nelvagal, H.R., Gore, K., Clemo, N.K., Waddington, S.N., and Rahim, A.A. (2024). Comparison of different promoters to improve AAV vector-mediated gene therapy for neuronopathic Gaucher disease. Hum. Mol. Genet. *33*, 1467–1480. https://doi.org/10.1093/hmg/ddae081.

38. Shen, S., Bryant, K.D., Brown, S.M., Randell, S.H., and Asokan, A. (2011). Terminal n-linked galactose is the primary receptor for adeno-associated virus. J. Biol. Chem. *286*, 13532–13540. https://doi.org/10.1074/jbc.M110.210922.

39. Hudry, E., Aihara, F., Meseck, E., Mansfield, K., McElroy, C., Chand, D., Tukov, F.F., and Penraat, K. (2023). Liver injury in cynomolgus monkeys following intravenous

and intrathecal scAAV9 gene therapy delivery. Mol. Ther. *31*, 2999–3014. https://doi.org/10.1016/j.ymthe.2023.07.020.

40. Bedoyan, J.K., Hage, R., Shin, H.K., Linard, S., Ferren, E., Ducich, N., Wilson, K., Lehman, A., Schillaci, L.A., Manickam, K., et al. (2020). Utility of specific amino acid ratios in screening for pyruvate dehydrogenase complex deficiencies and other mitochondrial disorders associated with congenital lactic acidosis and newborn screening prospects. JIMD Rep. *56*, 70–81. https://doi.org/10.1002/jmd2.12153.

41. Piotrowski, D., Clemensson, E.K.H., Nguyen, H.P., and Mark, M.D. (2024). Phenotypic analysis of ataxia in spinocerebellar ataxia type 6 mice using DeepLabCut. Sci. Rep. *14*, 8571. https://doi.org/10.1038/s41598-024-59187-0.

42. Lacava, S.A., Isilak, N., and Uusisaari, M.Y. (2024). The role of mouse tails in response to external and self-generated balance perturbations on the roll plane. J. Exp. Biol. *227*, jeb247552. https://doi.org/10.1242/jeb.247552.

43. Takayanagi, N., Beppu, H., Mizutani, K., Tomita, Y., Nagao, S., Suzuki, S., Orand, A., Takahashi, H., and Sonoda, S. (2013). Pelvic axis-based gait analysis for ataxic mice. J. Neurosci. Methods *219*, 162–168. https://doi.org/10.1016/j.jneumeth.2013.07.011.

44. Amende, I., Kale, A., McCue, S., Glazier, S., Morgan, J.P., and Hampton, T.G. (2005). Gait dynamics in mouse models of Parkinson's disease and Huntington's disease. J. Neuroeng. Rehabil. *2*, 20. https://doi.org/10.1186/1743-0003-2-20.

45. Findley, T.M., Wyrick, D.G., Cramer, J.L., Brown, M.A., Holcomb, B., Attey, R., Yeh, D., Monasevitch, E., Nouboussi, N., Cullen, I., et al. (2021). Sniff-synchronized, gradient-guided olfactory search by freely moving mice. Elife *10*, e58523. https://doi.org/10.7554/eLife.58523.

46. Maitland, A.D., Gonzalez, N.R., Walther, D., Pereira, F., Baumann, M.H., and Glatfelter, G.C. (2025). Rapid, open-source, and automated quantification of the head twitch response in C57BL/6J mice using DeepLabCut and Simple Behavioral Analysis. Preprint at bioRxiv. https://doi.org/10.1101/2025.04.28.650242.

47. Zhang, H., Yang, B., Mu, X., Ahmed, S.S., Su, Q., He, R., Wang, H., Mueller, C., Sena-Esteves, M., Brown, R., et al. (2011). Several rAAV vectors efficiently cross the blood-brain barrier and transduce neurons and astrocytes in the neonatal mouse central nervous system. Mol. Ther. *19*, 1440–1448. https://doi.org/10.1038/mt.2011.98.

48. Buckner, R.L. (2013). The cerebellum and cognitive function: 25 years of insight from anatomy and neuroimaging. Neuron *80*, 807–815. https://doi.org/10.1016/j.neuron.2013.10.044.

49. Pardridge, W.M. (2005). The blood-brain barrier: Bottleneck in brain drug development. NeuroRx. *2*, 3–14. https://doi.org/10.1602/neurorx.2.1.3.

50. Johanson, C.E. (1980). Permeability and vascularity of the developing brain: Cerebellum vs cerebral cortex. Brain Res. *190*, 3–16. https://doi.org/10.1016/0006-8993(80)91155-5.

51. Bunuales, M., Garduno, A., Chillon, M., Bosch, A., Gonzalez-Aparicio, M., Espelosin, M., Garcia-Gomara, M., Rico, A.J., Garcia-Osta, A., Cuadrado-Tejedor, M., et al. (2024). Characterization of brain transduction capability of a BBB-penetrant AAV vector in mice, rats and macaques reveals differences in expression profiles. Gene Ther. *31*, 455–466. https://doi.org/10.1038/s41434-024-00466-w.

52. Gholizadeh, S., Tharmalingam, S., MacAldaz, M.E., and Hampson, D.R. (2013). Transduction of the central nervous system after intracerebroventricular injection of adeno-associated viral vectors in neonatal and juvenile mice. Hum. Gene Ther. Methods *24*, 205–213. https://doi.org/10.1089/hgtb.2013.076.

53. Semple, B.D., Blomgren, K., Gimlin, K., Ferriero, D.M., and Noble-Haeusslein, L.J. (2013). Brain development in rodents and humans: Identifying benchmarks of maturation and vulnerability to injury across species. Prog Neurobiol *106–107*, 1–16. https://doi.org/10.1016/j.pneurobio.2013.04.001.

54. Salabarria, S.M., Corti, M., Coleman, K.E., Wichman, M.B., Berthy, J.A., D'Souza, P., Tifft, C.J., Herzog, R.W., Elder, M.E., Shoemaker, L.R., et al. (2024). Thrombotic microangiopathy following systemic AAV administration is dependent on anti-capsid antibodies. J. Clin. Invest. *134*, e173510. https://doi.org/10.1172/JCI173510.

55. Kim, J.Y., Grunke, S.D., Levites, Y., Golde, T.E., and Jankowsky, J.L. (2014). Intracerebroventricular viral injection of the neonatal mouse brain for persistent and widespread neuronal transduction. J. Vis. Exp. *91*, 51863. https://doi.org/10.3791/51863.

56. Karda, R., Rahim, A.A., Wong, A.M.S., Suff, N., Diaz, J.A., Perocheau, D.P., Tijani, M., Ng, J., Baruteau, J., Martin, N.P., et al. (2020). Generation of light-producing somatic-transgenic mice using adeno-associated virus vectors. Sci. Rep. *10*, 2121. https://doi.org/10.1038/s41598-020-59075-3.

57. Massaro, G., Mattar, C.N.Z., Wong, A.M.S., Sirka, E., Buckley, S.M.K., Herbert, B.R., Karlsson, S., Perocheau, D.P., Burke, D., Heales, S., et al. (2018). Fetal gene therapy for neurodegenerative disease of infants. Nat. Med. *24*, 1317–1323. https://doi.org/10.1038/s41591-018-0106-7.

OMTA, Volume *34*

Supplemental information

Next generation AAV-F capsid gene therapy rescues disease pathology in a model of pyruvate dehydrogenase complex deficiency

Anna Keegan, Özge Çetin, Ellie M. Chilcott, Juan Antinao Diaz, Simon Eaton, Simon N. Waddington, John R. Counsell, Shamima Rahman, and Rajvinder Karda

Figure S1

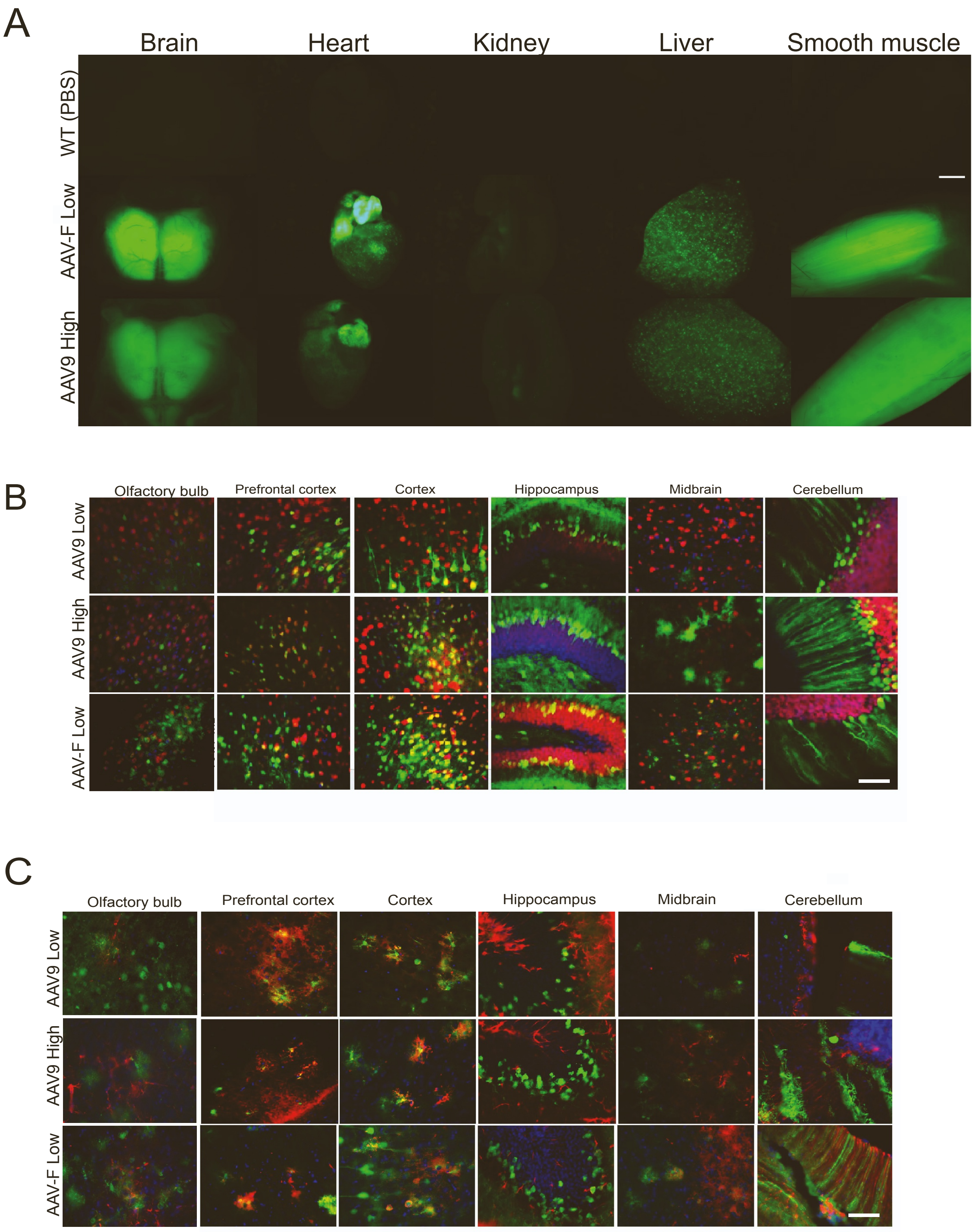

Figure S1. GFP Biodistribution of AAV9 and AAV-F. (A) Representative images of *ex vivo* GFP after neonatal ICV injections of AAV-F and AAV9. *Ex-vivo* GFP analysis at 100 days post injection. Scale bars shown in each image (2mm). (B) Transduction of neurons following the administration of AAV9 low dose and high dose and AAV-F low dose. Co-immunofluorescence with neuronal marker NeuN (red), GFP (green) and nuclear stain, DAPI (blue). Representative images of olfactory bulb, prefrontal cortex, cortex (reused from Figure 1D), hippocampus, midbrain and cerebellum. Images were taken at 40X magnification. Scale bar = 100μm. (C) Transduction of astrocytes following the administration of AAV9 low dose and high dose and AAV-F low dose. Co-immunofluorescence with astrocyte marker GFAP (red), GFP (green) and nuclear stain, DAPI (blue). Representative images of olfactory bulb, prefrontal cortex, cortex (reused from Figure 1F), hippocampus, midbrain and cerebellum. Images were taken at 40X magnification. Scale bar = 100μm.

Figure S2

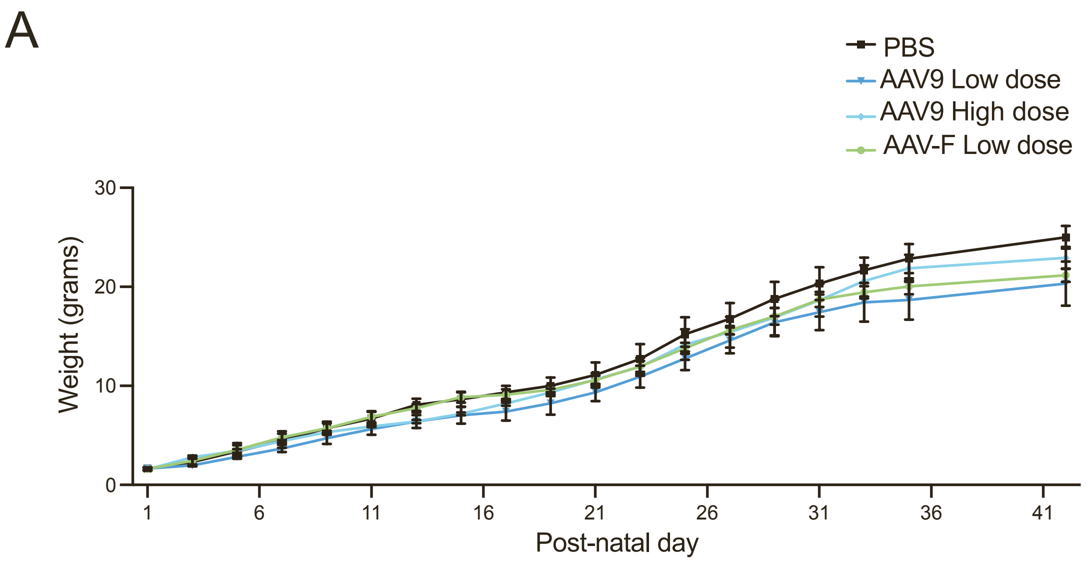

Figure S2. WT toxicology study of therapeutic vector following ICV delivery of AAV9 and AAV-F. (A) Weights of WT treated mice shown as mean ± SEM. One-way ANOVA multiple comparison.

Figure S3

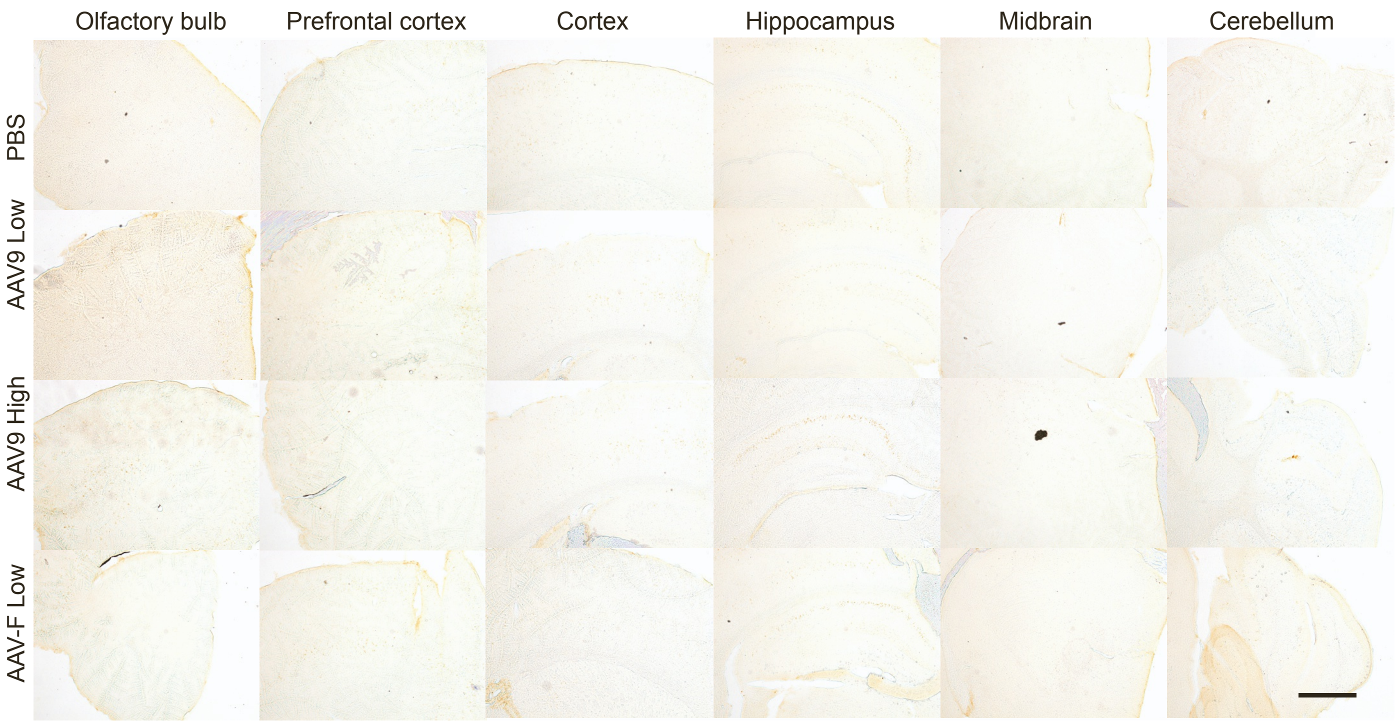

Figure S3. CD68 immunohistochemical assessment of treated WT brain samples. Representative images of CD68 expression in WT mice treated with PBS, AAV9 low dose, AAV9 high dose and AAV-F low dose for prefrontal cortex, cortex, hippocampus, midbrain and cerebellum. Images were taken at 40X magnification. Scale bar = 100µm.

Figure S4

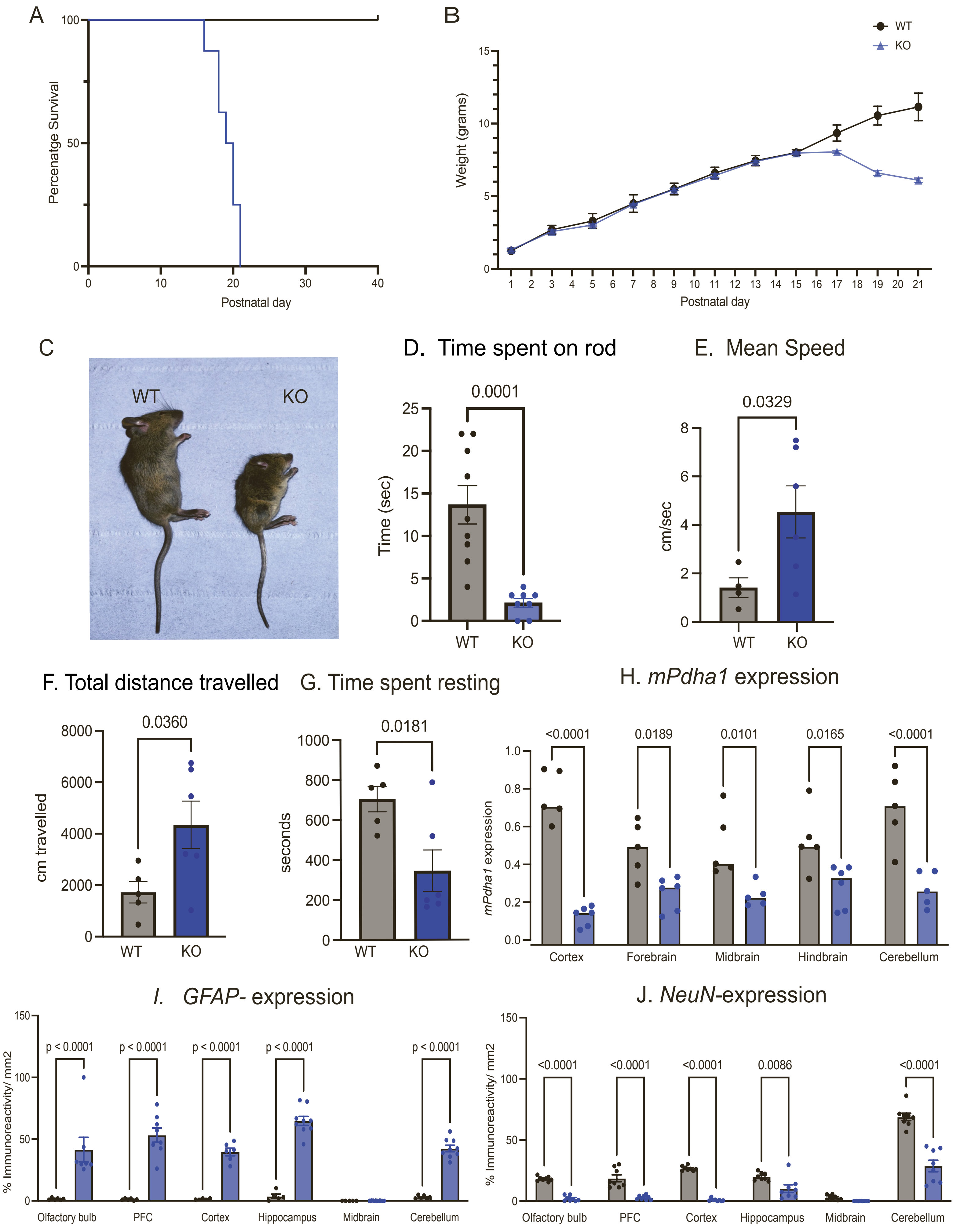
A
Percenatge Survival
Postnatal day
B
WT
KO
Weight (grams)
Postnatal day
C
WT
KO
D. Time spent on rod
0.0001
Time (sec)
WT
KO
E. Mean Speed
0.0329
cm/sec
WT
KO
F. Total distance travelled
0.0360
cm travelled
WT
KO
G. Time spent resting
0.0181
seconds
WT
KO
H. mPdha1 expression
<0.0001
0.0189
0.0101
0.0165
<0.0001
mPdha1 expression
Cortex
Forebrain
Midbrain
Hindbrain
Cerebellum
I. GFAP- expression
p < 0.0001
% Immunoreactivity/ mm2
Olfactory bulb
PFC
Cortex
Hippocampus
Midbrain
Cerebellum
J. NeuN-expression
<0.0001
0.0086
% Immunoreactivity/ mm2
Olfactory bulb
PFC
Cortex
Hippocampus
Midbrain
Cerebellum

Figure S4

Figure S4. Characterisation of untreated PDHc deficient mice. (A) Kaplan-Meier survival curve of KO mice compared to healthy controls (n=10). (B) Weights of KO mice compared to healthy controls. (C) Image of PDHc deficient mouse (right) alongside WT litter mate (left). (D) Rotarod assessment plotted as time (seconds) spent on the accelerating rod. *Pdha1* KO mice had a significantly lower latency to fall compared to healthy age matched littermates at postnatal 16. (E) Open field test at postnatal 16 showing mean speed over 15 minutes. Welch's t-test ± SEM. (n=10). (F) Open field test showing total distance travelled in 15 minutes. Welch's t-test ± SEM. (G) Open field test quantifying the resting time in 15 minutes. Welch's t-test ± SEM. (H) Fold change of *Pdha1* gene expression to *Gapdh* in *Pdha1* KO mice compared to WT littermates at time of collection (P18-21). (I) GFAP expression in *Pdha1* KO brain compared to WT littermates showing an upregulation of astrocytes throughout the brain. (J) NeuN expression in *Pdha1* KO brain compared to WT littermates showing extensive neuronal loss.

Figure S5

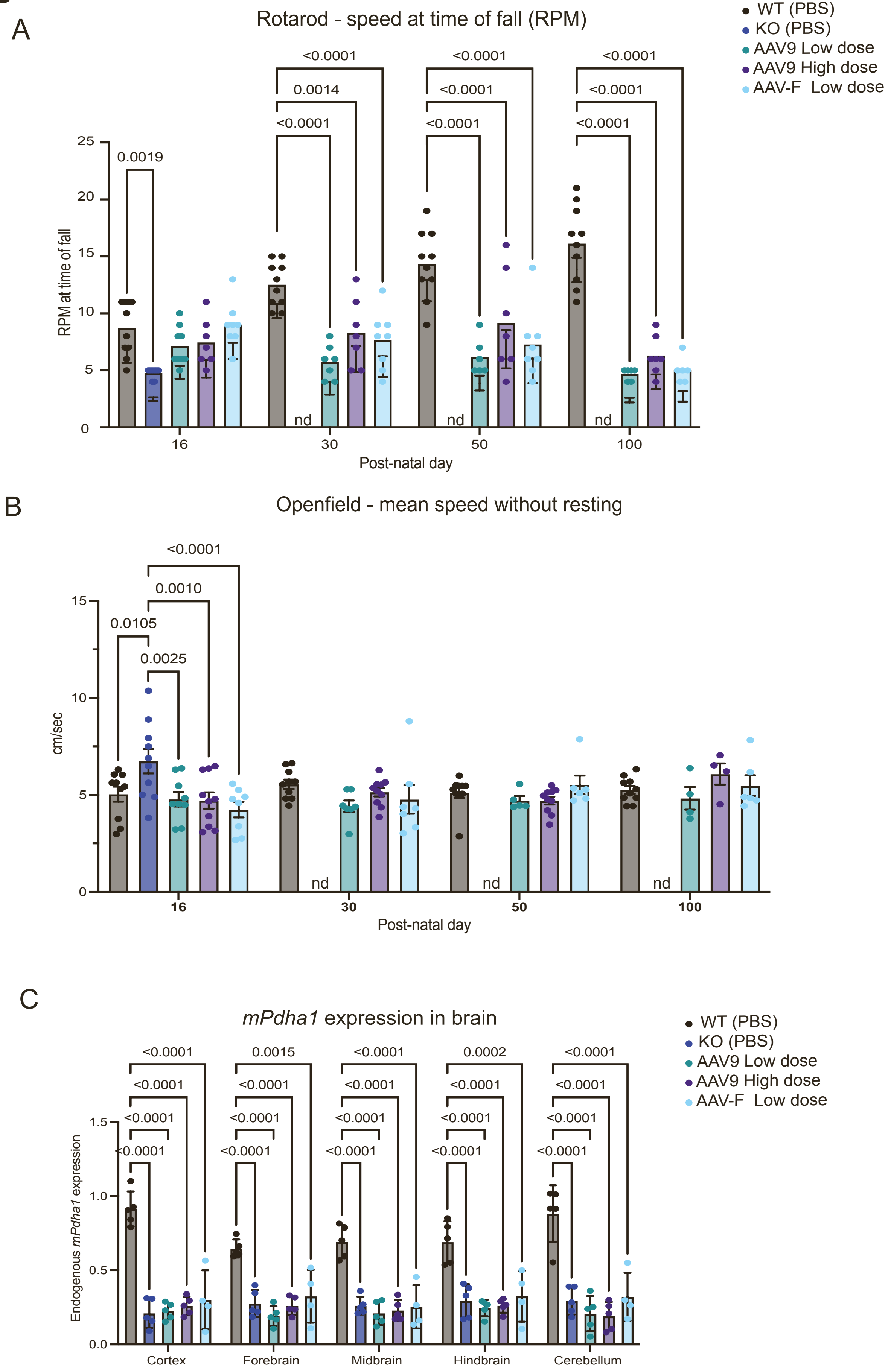
A
Rotarod - speed at time of fall (RPM)
WT (PBS)
KO (PBS)
AAV9 Low dose
AAV9 High dose
AAV-F Low dose
RPM at time of fall
0.0019
<0.0001
0.0014
<0.0001
nd
Post-natal day
16
30
50
100
B
Openfield - mean speed without resting
cm/sec
0.0105
0.0025
0.0010
<0.0001
C
mPdha1 expression in brain
Endogenous mPdha1 expression
0.0015
0.0002
Cortex
Forebrain
Midbrain
Hindbrain
Cerebellum

Figure S5

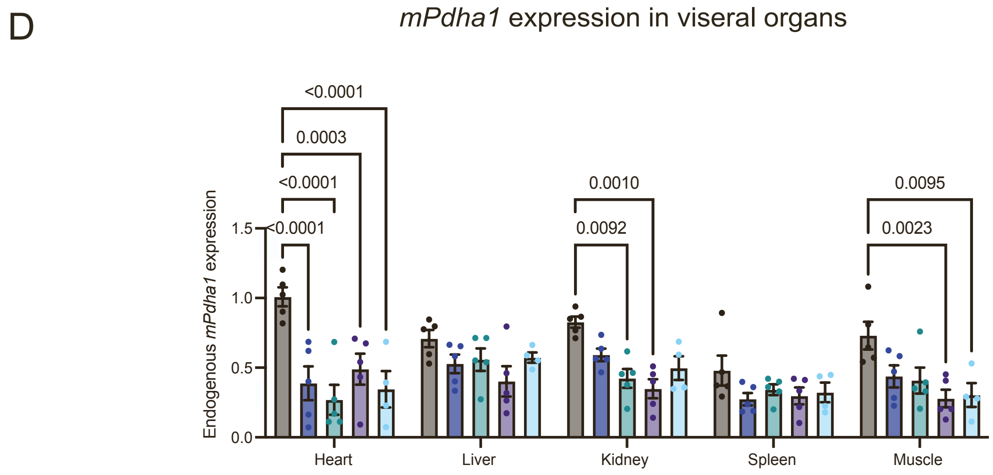

Figure S5. Further assessment of PBS-treated WT controls, and PBS, AAV9 low dose and high dose and AAV-F low dose treated KO's. (A) Behavioural rotarod assessment, showing speed on the time of fall. One-way ANOVA, Tukey's multiple comparison ± SEM. Not done (nd). (B) Open field test showing mean speed without resting in 15 minutes. One-way ANOVA, Tukey's multiple comparison ± SEM. Not done (nd). (C) Endogenous *Pdha1* expression in discrete regions of the brains of the treated KO mice. One-way ANOVA, Tukey's multiple comparison ± SEM. (D) Endogenous *Pdha1* expression in visceral organs of the treated KO mice. One-way ANOVA, Tukey's multiple comparison ± SEM.

Figure S6

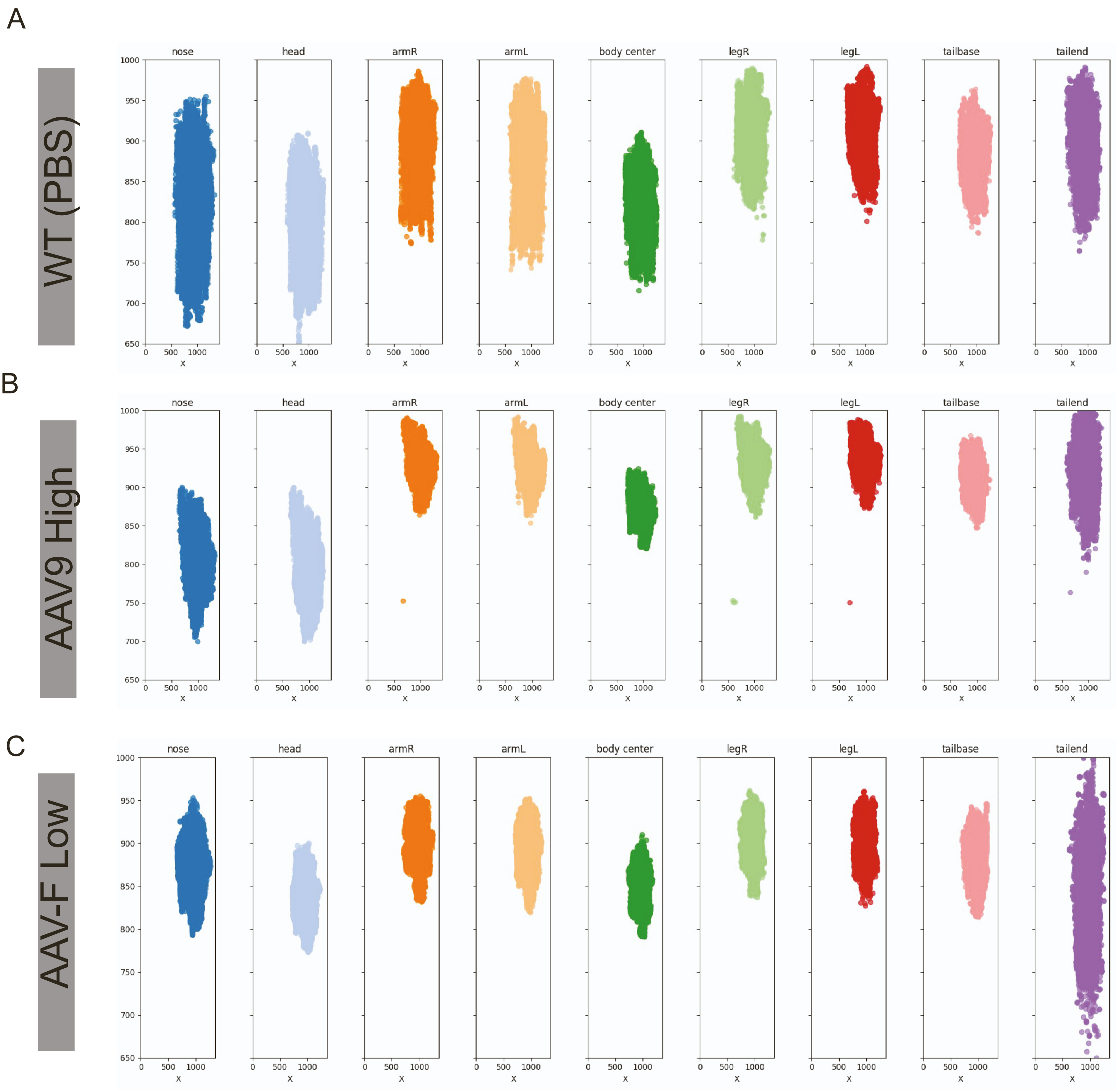

Figure S6. Individual body part assessment using DLC. (A) This plot visualises the movement path of a specific body part across time. Each point represents the X–Y position in the video frame, allowing quantification of locomotor patterns and spatial exploration. Targeted body part separated trajectory plots representing the PBS-treated control group. In all graphs, each body part is consistently color-coded as follows: nose (blue), head (light blue), right arm (dark orange), left arm (light orange), body centre (dark green), right leg (light green), left leg (red), tail base (pink), and tail end (purple). (B) Targeted body part separated trajectory plots representing AAV9 high dose treated KO mice show a change in individual body part movements compared to control mice. (C) Targeted body part separated trajectory plots representing AAV-F low dose treated KO mice show a change in individual body part movements compared to control mice.

Figure S7

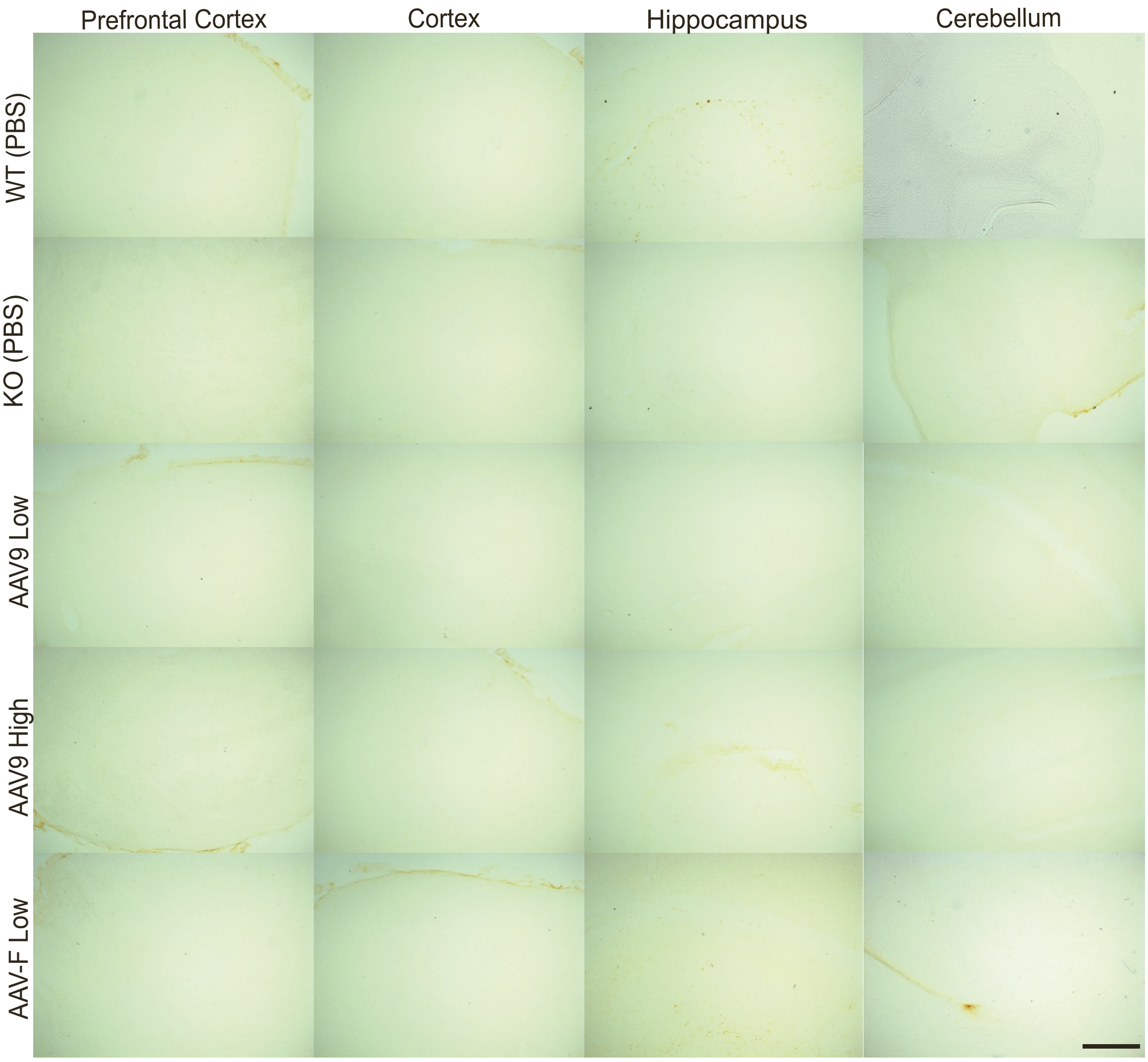

Figure S7. CD68 immunohistochemical assessment of treated brain samples. Representative images of CD68 expression in KO-treated mice for prefrontal cortex, cortex, hippocampus, and cerebellum. Images were taken at 40X magnification. Scale bar = 100μm.

Supplemental Methods

DLC Code

```
position_changes = {}
for group, dfs in group_data.items():
position_changes[group] = {}
for part in body_parts:
change_list = []
for df in dfs:
# Check if both x and y columns for the body part exist
if (part, 'x') in df.columns and (part, 'y') in df.columns:
x_series = df[(part, 'x')]
y_series = df[(part, 'y')]
# Keep only valid (non-zero) x and y values
valid_mask = (x_series != 0) & (y_series != 0)
x_valid = x_series[valid_mask]
y_valid = y_series[valid_mask]
# Need at least 2 valid points to compute displacement
if len(x_valid) >= 2 and len(y_valid) >= 2:
dx = np.diff(x_valid)
dy = np.diff(y_valid)
step_distances = np.sqrt(dx**2 + dy**2)
total_movement = np.sum(step_distances)
change_list.append(total_movement)
else:
print(f"Not enough valid data for {part} in a file from group {group}.")
else:
print(f"Missing columns {(part, 'x')} or {(part, 'y')} in a file from group {group}.")
if change_list:
position_changes[group][part] = change_list
```

Supplemental Tables

Table S1. Table of primers, probes and sequences used in this study.

**Primer Target**		**Sequence 5'-3'**
mPdha1 Genotyping	For	CGT CTG TTGAGA GAG CAG CA
mPdha1 Genotyping	Rev	CGC ACA AGATAT CCA TTC CA
hGFAP-Cre Genotyping	For	ACT CCT TCATAA AGC CCT
hGFAP-Cre Genotyping	Rev	ATC ACT CGT TGC ATC GAC CG
hPDHA1	For	GGAGGATGGGCTCAAATACTAC
hPDHA1	Rev	GACCATCACACAAGTGACAGA
hPDHA1	Probe	ACGCCGAATGGAGTTGAAAGCAGA
mPdha1	For	ATTCCTGGACTCAGGGTAGAT
mPdha1	Rev	CCCTTACCAGACCTGCAATAG
mPdha1	Probe	TATCTTGTGCGTCCGAGAGGCAAC
mGapdh	For	ACGGCAAATTCAACGGCAC
mGapdh	Rev	TAGTGGGGTCTCGCTCCTGG
mGapdh	Probe	TTGTCATCAACGGGAAGCCCATCA

Supplemental Videos

Video S1. Videos of treated KO *Pdha1* mice labeled with body parts: Control mouse.

Video S2. Videos of treated KO Pdha1 mice labeled with body parts: AAV9 high dose treated mouse.

Video S3. Videos of treated KO Pdha1 mice labeled with body parts: AAV-F low dose treated mouse.
